# The infection cycle of the haloarchaeal virus HFTV1 is tightly regulated and strongly inhibits motility of its host

**DOI:** 10.1128/msystems.00704-25

**Published:** 2025-09-22

**Authors:** Sabine Schwarzer, Leonard E. Bäcker, Jeroen G. Nijland, Ismail Hayani Aji, Anne de Jong, Cristina Moraru, Claudia Steglich, Tessa E. F. Quax

**Affiliations:** 1 Department of Molecular Microbiology, Groningen Biomolecular Sciences and Biotechnology Institute, University of Groningen 3647, Groningen, the Netherlands; 2 Archaeal virus-host interactions, Faculty of Biology, University of Freiburg 9174, Freiburg, Germany; 3 Environmental Metagenomics, Faculty of Chemistry, Research Center One Health Ruhr of the University Alliance Ruhr, University Duisburg-Essen, Essen, Germany; 4 Department of Molecular Genetics, Biomolecular Sciences and Biotechnology Institute, University of Groningen 3647, Groningen, the Netherlands; 5 Genetics & Experimental Bioinformatics, Faculty of Biology, University of Freiburg 9174, Freiburg, Germany; Institute of Biochemistry and Biophysics of the Polish Academy of Sciences, Warsaw, Poland

**Keywords:** haloarchaea, archaeal virus, transcriptomics, infection cycle, virus-host interaction, gene expression profiling, differential RNA-seq, motility

## Abstract

Although viruses have been shown to infect all domains of life, our understanding of the genetic program behind the exploitation of host resources to produce progeny virions is thus far limited to several bacterial viruses. Therefore, to elucidate the transcriptome of euryarchaeal viruses and their hosts, we employed RNAseq analysis of samples taken at different time points from *Haloferax gibbonsii* LR2-5 cultures infected with the lytic model virus Haloferax tailed virus 1 (HFTV1). While following the transcription of viral genes throughout the infective life cycle, we observed a tight temporal regulation of viral transcripts and differential expression from within viral gene clusters. Furthermore, antisense RNAs (asRNAs) appear to play an important role in support of the timing of late-expressed viral genes. Therefore, with many differentially expressed transcripts, including intragenic transcripts and asRNAs, the regulatory machinery employed by HFTV1 contrasts with that of viral model systems (based on phages), in which antitermination and/or alternative polymerases (seemingly lacking in HFTV1) are more widespread. When examining differentially expressed host genes, we observed a strong downregulation of genes involved in motility, such as those encoding the archaellum and chemotaxis machinery, which was confirmed by swimming assays of HFTV1 infected cells. This might be a strategy of the virus to redirect energy flowing into movement toward the production of virions. In conclusion, this work provides a stepping stone for further exploration of the intriguing strategies of viral transcriptional regulation throughout their infection cycle across the domains of life.

**IMPORTANCE:**

Viruses infect members of all three domains of life, including *Archaea. Euryarchaea* are widespread microorganisms found in various environments, such as the human gut and solar salterns. Due to the exceptional availability of cell biology and genetic tools for some salt-loving archaea, they serve as a model system from which insights can be extrapolated. Insights into the regulation of viral infections are of particular importance, especially since HFTV1 has been adopted as a model virus by the archaeal viral community. We found that, while harboring parallels with bacterial viruses, such as tight temporal regulation, HFTV1 harbors an impressive number of differentially expressed transcriptional units. Furthermore, antisense RNAs and intragenic regulatory elements seem to play a much more prominent role in HFTV1 gene expression. Thus, this work challenges current models and provides valuable new insights into the gene regulation of viral infection of archaea, which mark similarities and differences with viruses from other domains of life.

## INTRODUCTION

Viral infections have been a continuous contributor to evolution and have brought forward countless host adaptations (1–5). Archaea, which are a diverse group of microorganisms that are more related to eukaryotes than to bacteria, have likewise been found to be preyed upon by viruses. These viruses are characterized by a high diversity of their genome content and viral capsids (6). The study of archaeal viral diversity and infection mechanisms has been important to draw hypotheses about the origin and evolutionary trajectory of viruses in general (6). Research on archaeal viruses lags behind our understanding of eukaryotic and bacterial viruses, due to the extreme conditions in which some hosts thrive and the lack of well-developed, genetically accessible model virus-host systems for archaea (6). For example, details of transcriptional regulation of infection are not available for most archaeal viruses. To bridge this knowledge gap, we selected *Haloferax gibbonsii* LR2-5 and its virus Haloferax tailed virus 1 (HFTV1), as these are a model for the study of virus-host interactions in *Euryarchaea* (7). *H. gibbonsii* LR2-5 and HFTV1 were isolated together from the hypersaline Lake Retba in Senegal (8). Besides HFTV1, nine other haloarchaeal viruses were found to infect *H. gibbonsii* LR2-5, making it a versatile haloarchaeal viral host (9). *H. gibbonsii* LR2-5 is closely related to *H. volcanii*. Whereas the *H. volcanii* genome contains several antiviral defense systems, such as CRISPR-Cas, the *H. gibbonsii* genome is almost completely devoid of known defense systems (7). This could be a possible reason for its high viral susceptibility compared to *H. volcanii* (9).

*H. gibbonsii* LR2-5 displays motility behavior in liquid medium and on semi-solid agar plates (7). Cells swim forward and reverse with the help of the archaellum, the rotating motility structure of archaea. By adjusting their forward and backward runs, they can swim toward or away from environmental stimuli, which are sensed by the chemotaxis system. In addition, *H. gibbonsii* LR2-5 was also shown to display growth-phase dependent changes in cell shape, as reported for multiple haloarchaea (7, 10). It transforms from motile rod-shaped cells in the early exponential phase to non-motile disc-shaped cells in stationary phase. Due to its aerobic growth at moderate temperatures (~40°C), *H. gibbonsii* LR 2-5 is further well suited for light microscopy (7). Recently, we developed a genetic system based on deletion of the *pyrE* gene, which leads to uracil auxotrophy and allows for counter selection with 5-FOA (11). This system enables the generation of genomic deletion mutants and the expression of genes under the tryptophan-inducible promoter. In addition, the salt-stable version of GFP was shown to be functional in *H. gibbonsii* LR2-5 and allows for tagging of proteins to study their cellular localization (11). This combination of broad viral susceptibility, genetic tractability, and availability of molecular tools makes *H. gibbonsii* LR2-5 an ideal model system for studying euryarchaeal viruses.

HFTV1 belongs to the *Haloferuviridae*, which is part of the *Caudoviricetes*. It has a long non-contractile tail and a linear dsDNA genome, which has so far been annotated with 68 open reading frames (8). Previously, we demonstrated that HFTV1 binds to the cell surface of *H. gibbonsii* within several minutes (12). It uses one of the two S-layer glycoproteins, the main cell wall components, as a receptor. Binding can take place with both the capsid head and tail (12). Cryo-electron microscopy has shown that the capsid head of HFTV1 is decorated with turrets, which likely bind the S-layer (13). We hypothesized that binding with the capsid head is reversible and is followed by irreversible binding through the tail, leading to genome delivery (12, 13). HFTV1 is a lytic virus that leads to cell lysis 6 hours post-infection (p.i.). Further details of the progression of the infection cycle between the initial binding of HFTV1 to the cell surface and cell lysis 6 hours later are not known. We aimed to gain insight into the regulation of the genetic program behind intracellular progression of the HFTV1 infection and thus to shed light on the viral life cycle of euryarchaeal viruses. We explored the transcriptome of virus and host during infection with RNA sequencing (RNAseq). Therefore, this study provides valuable insights into the temporal regulation of the HFTV1 infection cycle and its impact on the viral host, which will pave the way for further studies on the intricacies of the HFTV1 life cycle.

## RESULTS AND DISCUSSION

**Fig 1:**
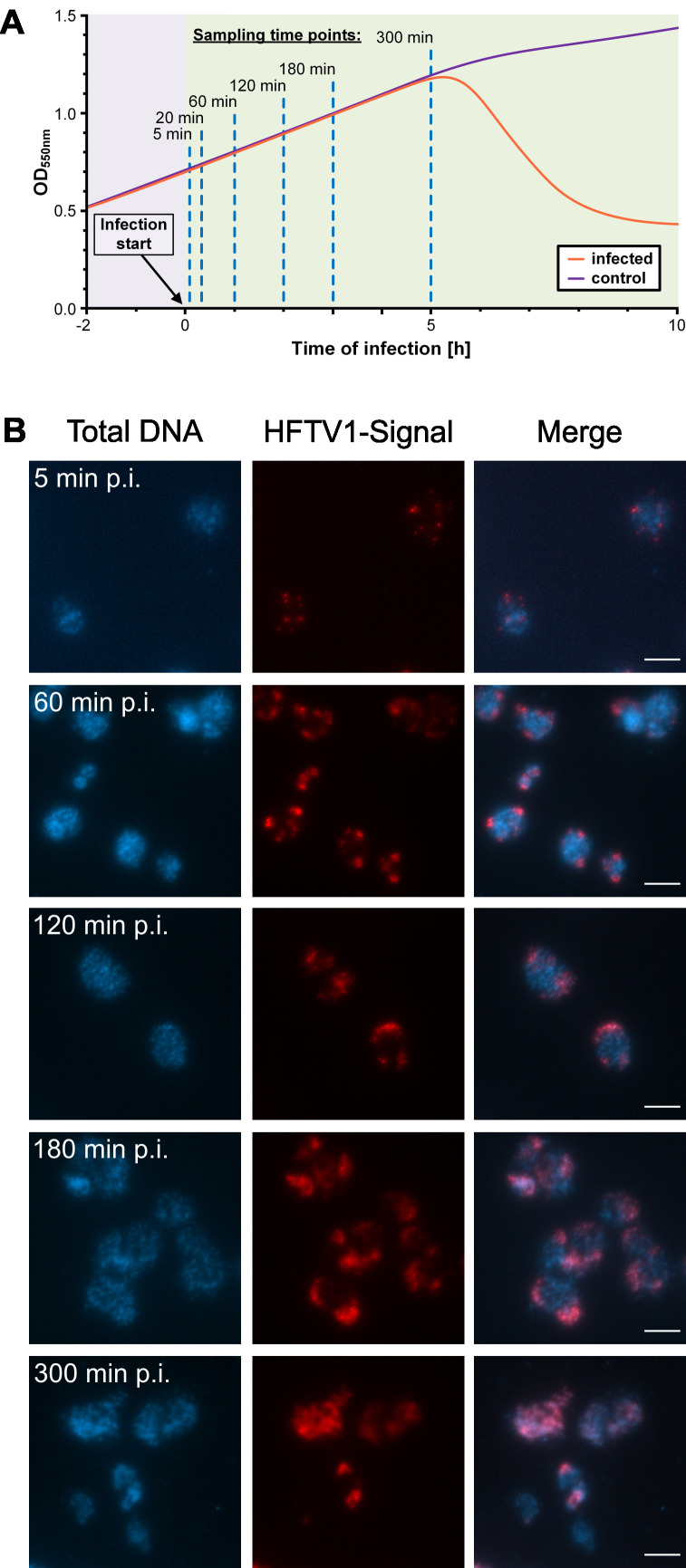
The infection cycle of HFTV1. (**A**) Example of growth dynamics during the course of HFTV1 infection over time. Infected *H. gibbonsii* LR2-5 host cultures increase in cell abundance until lysis occurs after 5 to 6 hours, and cell densities decrease. Samples for virus-targeted direct-gene FISH and RNA isolation were collected from infected and virus-free cultures at the indicated time points. (**B**) Fluorescence *in situ* hybridization of nucleic acids (virus-targeted direct-gene FISH). Visualization of the infection at M.O.I. of 10 of *H. gibbonsii* LR2-5 by HFTV1 using direct-gene FISH. The first column shows the total DNA content of cells visualized by DAPI staining. The second column shows attached and intracellular virus visualized by HFTV1-targeting probes labeled with Alexa594. The third column shows the merge of DAPI and virus signal. The scale bar indicates 5 µm.

To follow the progression of the HFTV1 infection, *H. gibbonsii* LR2-5 was infected, and samples were taken at relevant intervals after infection (Fig. 1A). To monitor the dynamics of the lytic virus life cycle, we performed virus-targeted fluorescence *in situ* hybridization (virus-targeted direct-gene FISH) using Alexa594-labeled probes specific to the HFTV1 genome (Table S1). A progressive increase in viral signal was observed from 5 min post-infection (p.i.) to 300 min p.i. (Fig. 1B). At early time points (5 min p.i.), single-dot signals, presumably corresponding to individual viral genomes, were detected, representing viral particles immediately after adsorption and injection. At 60–120 min after infection, an increase in fluorescence intensity was observed, indicating viral genome replication within the cells. The appearance of distinct fluorescent regions reflected the formation of viral assembly sites, indicating that the infection had reached a more advanced stage involving viral genome packaging and capsid assembly, which we refer to as viral maturation. Evidence of potential cell lysis events was observed at 300 min p.i., marked by a decline in cell population and an increase in cell debris, as supported by accompanying optical density (OD_600_) measurements indicating a loss of intact cells.

### Improved annotation of the HFTV1 genome reveals new ORFs and putative gene functions

To understand how the HFTV1 virus is affecting the *H. gibbonsii* LR2-5 gene expression levels and how the viral gene expression is regulated, RNA was isolated after 5, 20, 60, 120, 180, and 300 min p.i. cDNA libraries were prepared, subjected to Illumina sequencing, and reads were mapped to the viral and host genomes. On average, 10.1 × 10^6^ ± 0.93 ×10^6^ reads were analyzed of all samples of which ~87% align to the host genome at *T*_0_, which was reduced to ~24% after 300 min p.i. After 300 min p.i., ~71% of all reads align to the HFTV1 genome.

Since the original publication of the HFTV1 genome (8), new annotation tools and experimental data have become available (13). Therefore, to improve our ORF designations in the RNAseq read mapping, we updated the annotation of the HFTV1 genome and employed Pharokka (14) in conjunction with Phold (https://github.com/gbouras13/phold) to identify further ORFs and predict functions for genes of unknown purpose.

**Fig 2:**
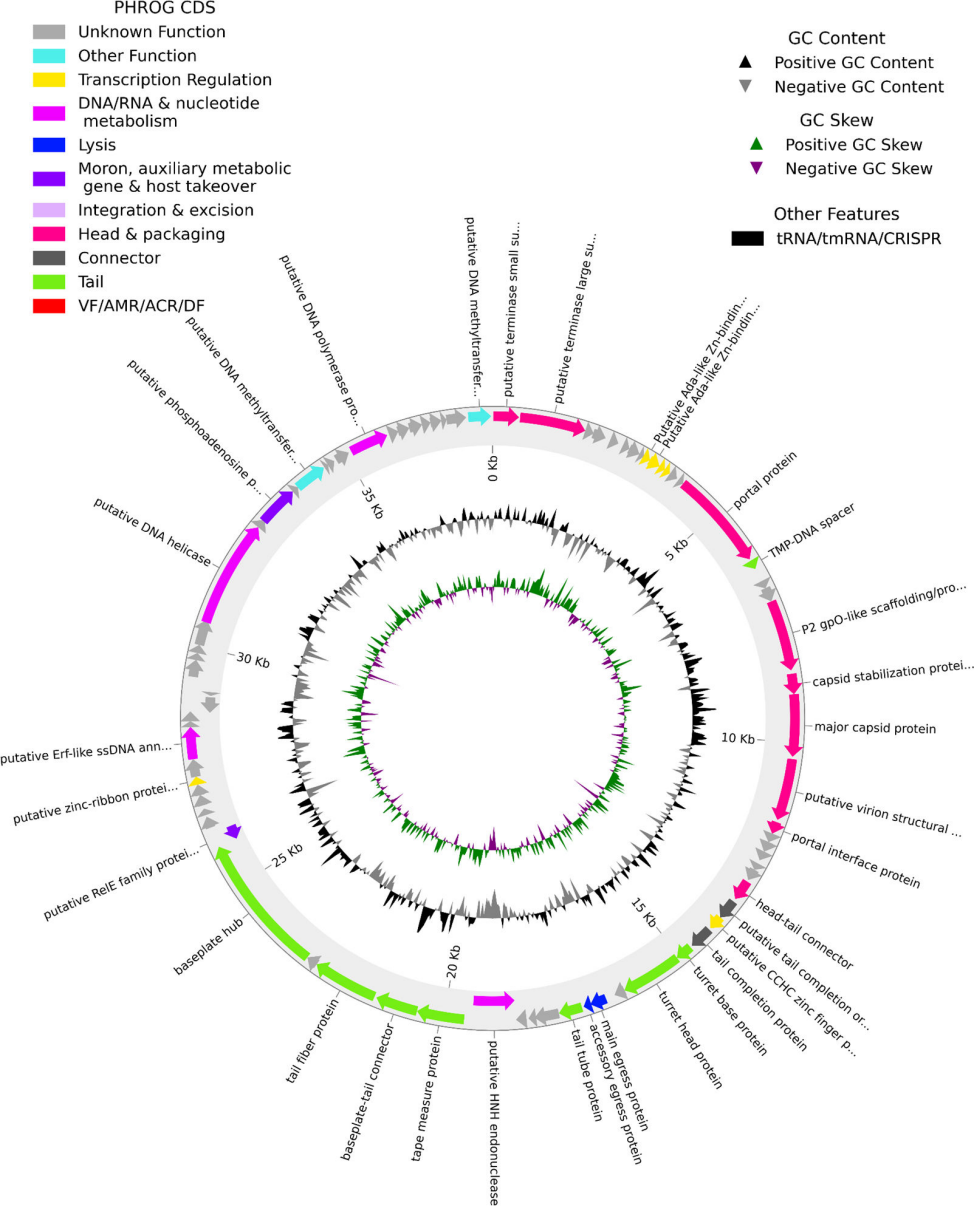
Updated annotation of the HFTV1 genome. The reference genome (NC_062739.1) was updated with Pharokka and Phold (see Materials and Methods) as well as with RNAseq. Different colors indicate genes of different predicted functional groups, based on their respective PHROG (prokaryotic virus remote homologous group) assignment. Genome map of the phage showing annotated open reading frames (ORFs) and functional modules. The genome is depicted in circular format for visualization purposes only. The actual phage genome is most likely linear and circularly permuted, consistent with the packaging mechanism inferred from sequence homology.

In this process, 12 further ORFs (80 total, formerly 68) and one putative tRNA were found in the HFTV1 genome. In addition, some conflicts between the original and the predicted new ORF assignment in the HFTV1 genome were identified. To resolve those, we used the RNAseq data (as described below). For instance, we found ORFs in the old annotation, which had start codons upstream of transcriptional start sites (TSSs), indicating that the actual start codon must be further downstream inside the transcribed region. Additionally, long untranslated regions (UTRs) pointed us to potentially overlooked ORFs. Similarly, because unidirectional genes within the same transcript generally rely on translational coupling via termination–reinitiation (15–17), we were confident that newly annotated genes within intergenic regions of a continuous transcript are likely to be correct. The resulting curated annotation is shown in Fig. 2 and is used for the alignment of the following HFTV1 RNAseq analysis.

### Expression of viral genes

**Fig 3:**
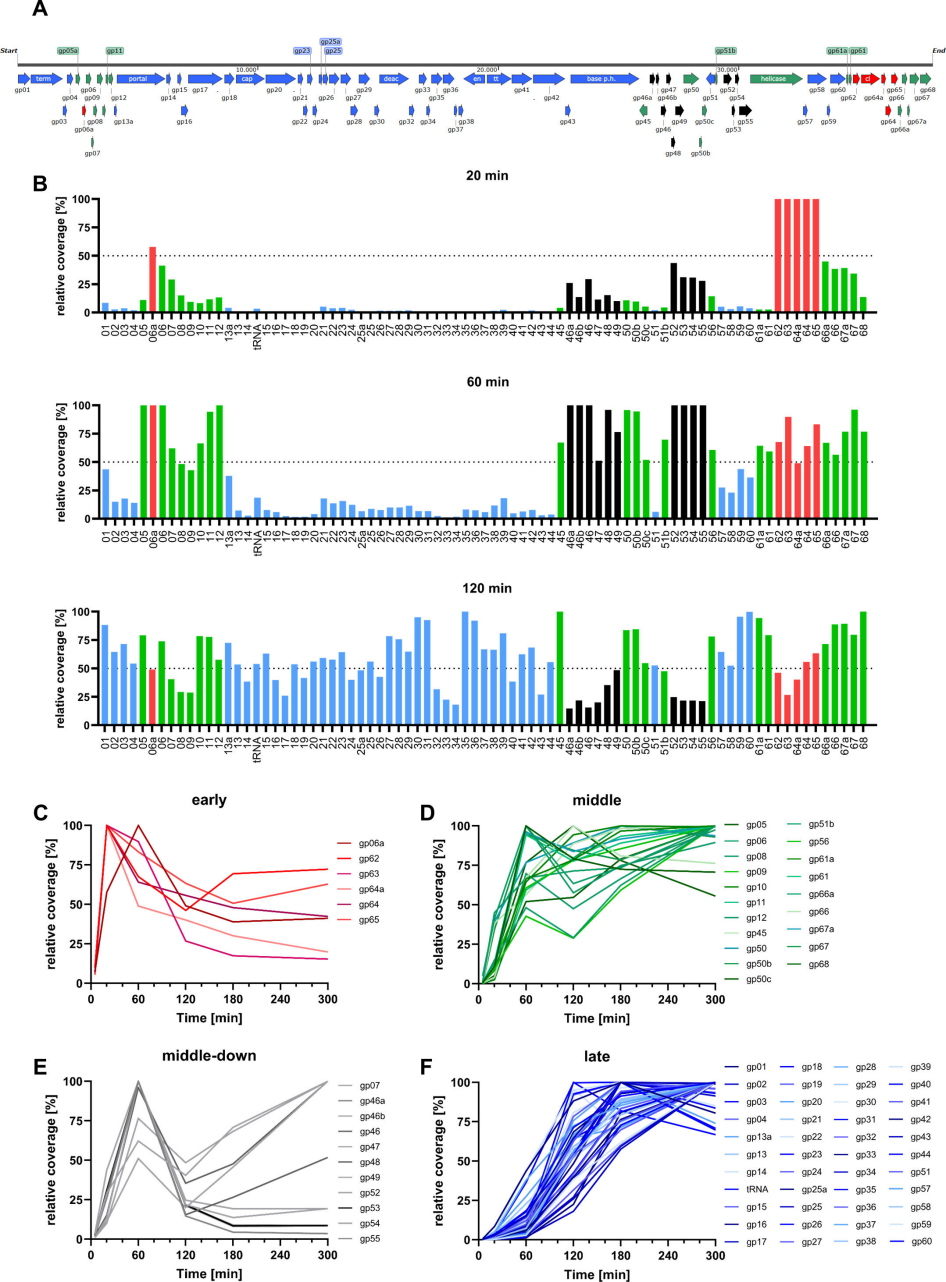
Expression of HFTV1 genes during viral infection of *H. gibbonsii* LR2-5. (**A**) Schematic representation of the newly annotated HFTV1 genome, with genes color-coded according to their expression timing: early (red), middle (green), middle-down (black), and late (blue). (**B**) Gene length-normalized gene expression profiles of HFTV1 at 20, 60, and 120 min post-infection. The *y*-axis represents the percentage of the total gene expression of the individual genes. The early, middle-down, middle, and late expressed gene groups are represented in red, black, green, and blue, respectively. (**C–F**) Total percentage coverages of viral ORFs over time for gene clustering into the respective expression groups introduced in panel **B**: (**C**) early, (**D**) middle-down, (**E**) middle, and (**F**) late. The results shown in panels** B–F** reflect the averages of three biological replicates.

To gain insights into the differential expression of HFTV1 throughout the infection, the HFTV1 reads were normalized for gene length using SeqMonk V1.48.1. Due to the short incubation time at time points 5 and 20 min p.i., only a small fraction of all reads align to the HFTV1 viral genome (as can be seen in the low coverages for these time points in the supplemental data set). However, this number of viral reads rapidly increases over time. Based on the temporal trend of reads mapping to protein-coding annotated genes (and one tRNA), we divided the HFTV1 genes into “early” (colored red), “middle” (colored green), “middle-down” (colored black), and “late” (colored blue) expression groups (Fig. 3A). For this classification, we assessed the gene expression at each time point by normalizing read coverage by gene length and total reads, followed by calculating percentage coverage relative to the maximum coverage for each gene across all six time points (calculated as RPKM_gpXX_ at *t*_*n*_/max [RPKM_gpXX_] × 100%). As shown in Fig. 3B, the grouping is then assigned based on whether genes reached their half-maximum expression (dotted line) by 20 min (“early”; 6/81; 7% total), 60 min (“middle”; 20/81; 25% total); or after 60 min (“late”; 44/81; 54% total). Furthermore, genes that reached their half-maximum expression by 60 min but later fell below this half-maximum threshold are referred to as “middle-down” genes (11/81; 14% total). The relative percentage coverage was also plotted over time for each gene of the respective temporal categories, showing the clustering into expression trends (Fig. 3C through F).

**Fig 4:**
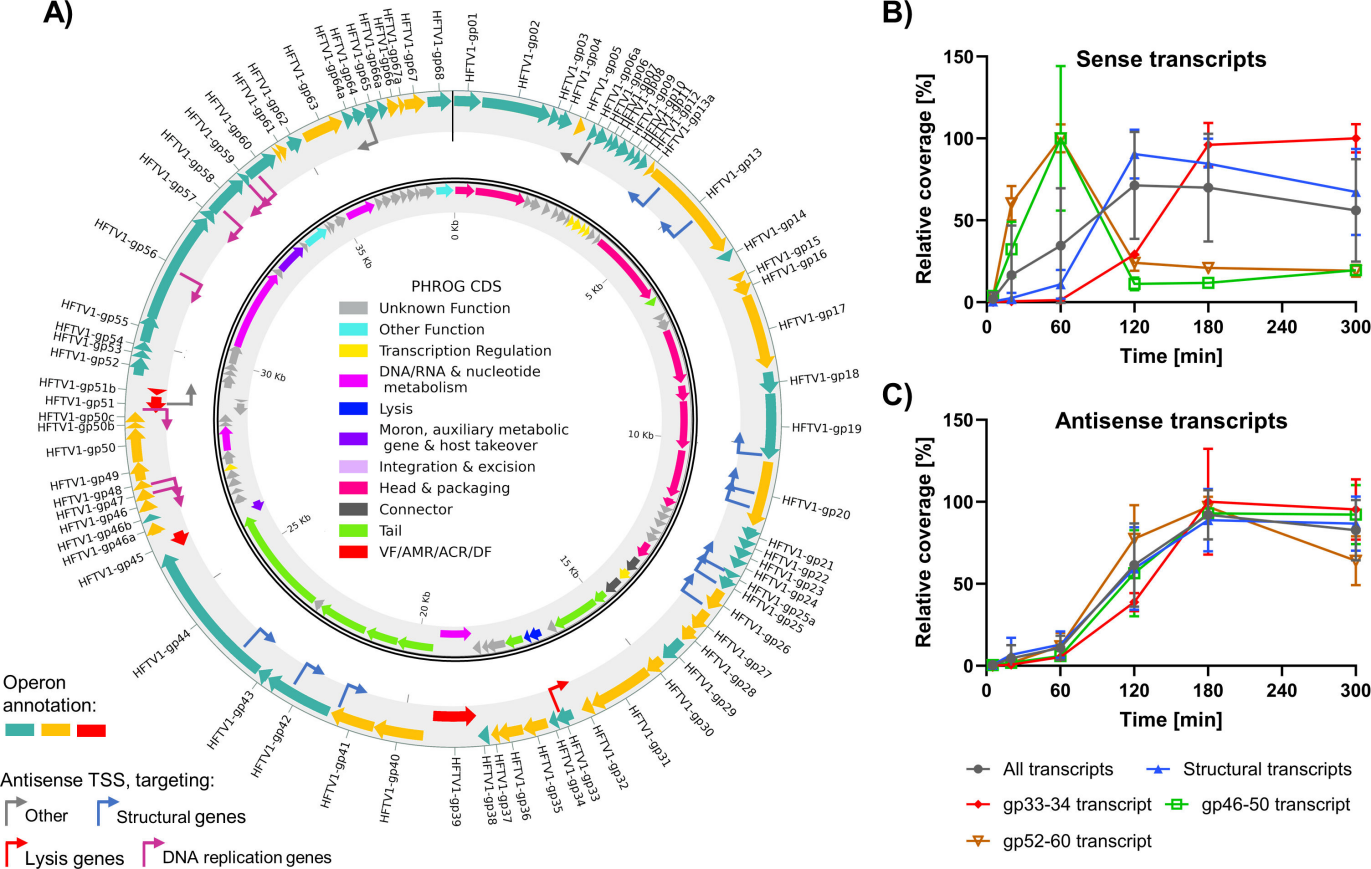
Transcriptional organization of the HFTV1 genome. (**A**) Outer ring: assignment of the HFTV1 ORFs into operon structures, indicated with alternating coloration of ochre and teal for forward operons and red for reverse operons. The bent arrows indicate identified antisense transcriptional start sites (TSS) and are colored according to the types of genes with which the corresponding antisense RNA would hybridize. Although not additionally annotated, every operon is preceded by a sense TSS. Inner ring: functional grouping of the HFTV1 genome (cfr. Fig. 2). (**B**) Relative percentage coverage over time of selected viral sense transcripts associated with antisense RNA interactions during the HFTV1 infective life cycle. (**C**) Relative percentage coverage over time of viral antisense transcripts during the HFTV1 infective life cycle. For panels **B** and **C**, error bars represent the averages and standard deviations of the coverages of multiple TSS, or, in case data of a single TSS is shown, averages and standard deviations among the tree replicates for the same data point.

A noteworthy feature of the HFTV1 expression is that some gene clusters that appear to be bona fide operons are differentially expressed. For example, gene cluster *gp52–gp60* appears to be a bona fide operon, based on its genomic layout (Fig. S1A). G*p52–gp60* seemed to be located downstream of a common promoter, and the genes have overlapping stop and start codons without intergenic sequences. However, the trend in their expression pattern over time is significantly different for various genes located in the same cluster. In the gene cluster *gp52–gp60, gp52–gp55* are “middle-down” genes, indicating that their onset of expression is in the middle of the infection cycle and subsequently is decreased severely (Fig. 3D; Fig. S1B), and *gp56–gp60* are “middle” to “late” genes, for which the read coverage does not decrease in late stages of infection (Fig. S1B). This pattern is indicative of intergenic promoters, which in *H. volcanii* have been shown to account for approximately 6% of all identified transcripts (18). Therefore, we manually annotated all transcriptional start sites (TSSs) in the RNAseq mapping, based on the typical sudden spike in coverage at exactly the same position in the genome (see starting positions of operons in Fig. 4A). Indeed, during this process, we discovered a potential intergenic promoter within gp56 (TSS at 31.839 nt), which explains why the genes of the supposed gp52-gp60 operon appear to follow two distinct temporal expression trends. Interestingly, based on coverage distribution, gp56 appears to be a middle gene. However, since the intergenic promoter is located within the ORF, the RNA coverage stemming from this internal TSS does not contribute to translatable gp56 mRNA. This finding effectively places gp56 in the middle-down (almost early) gp52–gp55 group, which, due to its hypothetical function in genome replication, also fits conceptually. This suggests that the two promoters of the gp52–gp60 region are differently regulated, which may be evidence of the high level of optimization in genome organization that has taken place in this virus to allow for maximum coding capacity while limiting genome size expansion.

A noteworthy feature of the HFTV1 expression is that some gene clusters that appear to be bona fide operons are differentially expressed. For example, gene cluster gp62–gp65 is expressed at an early onset, with gp63 (PCNA, DNA polymerase sliding clamp) being the only annotated gene. This is most likely required for viral DNA amplification, known as one of the first steps in the viral infection cycle (8). Additionally, many structural proteins, or proteins required for the assembly of the virus particle, are expressed in the late phase of infection, including gp13 (portal protein), gp17 (P2 gpO-like scaffolding/protease protein), gp18 (capsid stabilization protein), gp19 (HK97 gp5-like major capsid protein), gp29 (gp17-like tail completion protein), gp35 (tail tube protein), and gp40 (phage tail tape measure protein).

### Organization of the viral genome reveals clear antisense regulation of functional groups

As the transcriptional organization of the HFTV1 genome appears to be fairly complex and intergenic promoters may lead to incorrect assignment of the temporal expression group of genes (i.e., gp56), we proceeded to organize the HFTV1 ORFs into operons. We assigned genes into the same operon based on the following three criteria: (i) neighboring start-stop codons, (ii) clustering into the same temporal expression profiles (cfr. Fig. 3, with the exception of intergenic promoters), and (iii) a continuous coverage throughout the proposed transcript (Fig. 4A). The beginning of each operon must further be preceded by an identified TSS.

As a result, we clustered the 80 ORFs identified thus far into 28 distinctly regulated transcriptional units. Of these, 12 are monocistronic (42.8%), while the overall HFTV1 genome has an average of about three genes per operon. This is in stark contrast to phages, which generally have few but highly polycistronic operons (19–22). Therefore, the transcriptional organization of HFTV1 more closely mirrors that of its host. In *Haloferax volcanii*, about two-thirds of the transcripts are monocistronic, and thus, each operon, on average, contains a relatively small number of genes (1.7) (18). As such, the HFTV1 operon structure is intermediate between that of canonical phages and eukaryotic viruses, one of few instances where polycistronic operons can be found in this transcriptionally fundamentally different domain (23, 24).

Curiously, during the process of assigning HFTV1 reads to transcriptional units, we observed that in the case of gp20, gp33, gp44, gp47, gp48, gp50c, gp51, gp57, gp58, and gp59, at around three of the six sampling time points, more antisense reads mapped to these genes than sense reads. Therefore, we also identified the TSSs of these putative antisense RNA (asRNA) transcripts, which have been shown to play important regulatory roles in *Haloferax* species (18, 25, 26).

As shown in Fig. 4A, more than half (52.25%, 12/23) of these potential antisense transcripts (indicated as bend arrows) are located within or at the end of operons associated with structural genes involved in capsid formation and packaging. A second apparent hotspot for asRNA (30.48%, 7/23) is two operons containing genes associated with DNA replication. To better understand the potential mechanisms behind these observations, we quantified the transcription profiles of all identified promoters in the HFTV1 genome based on relative coverage at their respective TSSs over time and compared the temporal trends of the sense transcripts with their antisense counterparts (Fig. 4BC; Table S2). The average of all sense transcripts (Fig. 4A, gray line) does not show any temporal trend, indicating that genes with different temporal expression profiles (i.e., early or late) average out the coverage change over time. In contrast, the average of all antisense transcripts followed a “late” expression profile, peaking at around 180 min.

The sense transcripts mapping to genes of different functionalities show a very clear temporal pattern of early, mid, and late genes. We identified typical early functions, such as DNA metabolism (gp46–gp50 and gp52–gp60) peaking at 60 min p.i., structural genes that are highly expressed only from 120 min (mid genes), and finally the proposed (late) lysis transcript (gp33–gp34, Kuiper personal communication), which is only highly expressed from 180 min and reaching its maximum at 300 min, when host lysis occurs.

Therefore, the expression profiles of the identified HFTV1 transcripts appear to follow the typical viral life cycle progression patterns: from genome replication to virion production to egress (27–32). Interestingly, no alternative RNA polymerase has been identified in the HFTV1 genome, and no transcript elongation at later time points is evident (suggestive of antitermination) (Fig. 3B). Therefore, it appears that the regulation in the HFTV1 life cycle is accomplished by alternative mechanisms that control time-dependent transcription initiation regulation in conjunction with asRNA expression.

Occasionally, asRNA can also be found in phages, where it effectively silences the translation of the sense transcript (i.e., pOOP, paQ, or STnc6030 asRNA) (20, 33). Interestingly, similar to the above-mentioned examples of asRNA-mediated gene silencing, most identified antisense TSSs in the HFTV1 genome also have a relatively large distance (multiple kbs) to their respective sense TSS counterpart. Therefore, the late expression of nearly all asRNAs (such as those targeting the operons gp46–gp50 and gp52–gp60) suggests that they play an important role in downregulating the transcripts of genes involved in early infection functions, such as DNA metabolism. This is likely to shift all resources to particle maturation and egress. It is currently unclear why we also found asRNA in structural operons, but unlike operons of other functions, the sense read coverage for structural transcripts usually outnumbered the antisense reads (examples shown in Fig. S1C and S2), thus potentially being involved in fine-tuning rather than silencing translation.

Generally, it seems that asRNA-based regulation is much more involved in the life cycle regulation of HFTV1 than previously anticipated. However, this might also be based on methodological biases, as many studies on phages predate the availability of RNAseq and may have overlooked asRNAs. For instance, for the *Salmonella* phage BTP1, new asRNAs have recently been identified via RNAseq (33). Interestingly, one of these asRNAs (STnc6030), which is expressed during lysogeny, appears to mediate superinfection immunity by silencing a structural transcript while still permitting prophage induction (33).

### Impact of viral infection on host gene expression

Next, we analyzed how viral infection affects host gene expression. An intensity difference statistical analysis using DESeq2 with a *P*-value cutoff of 0.05 in SeqMonk (https://www.bioinformatics.babraham.ac.uk/projects/) of *H. gibbonsii* LR2-5 infected with HFTV1 showed 0, 7, 135, 247, 434, and 374 differentially expressed host genes at 5, 20, 60, 120, 180, and 300 min p.i., respectively, compared to the uninfected control culture. At earlier time points (20 and 60 min), the majority (100% and 88%, respectively) of the differentially expressed genes (DEGs) are upregulated, whereas later in the infection cycle, a more equal distribution between up- and downregulated genes is observed. Figure S3 illustrates the overlap and specificity of upregulated (Fig. S3A) and downregulated (Fig. S3B) DEGs at 5, 20, 60, 120, and 300 min p.i. While the majority of DEGs were up- or downregulated specific to individual time points, a small core set of upregulated genes (e.g., 33 genes; Fig. S3A) was shared across all time points starting from 60 min p.i. Similarly, a subset of 39 genes was consistently downregulated from 120 min onward. These patterns suggest a (possibly virus-mediated) host response to the viral infection by differentially regulating genes potentially involved in antiviral responses and, especially for mid-to-late infection stages, an apparent suppression of specific pathways in a coordinated manner.

**Fig 5:**
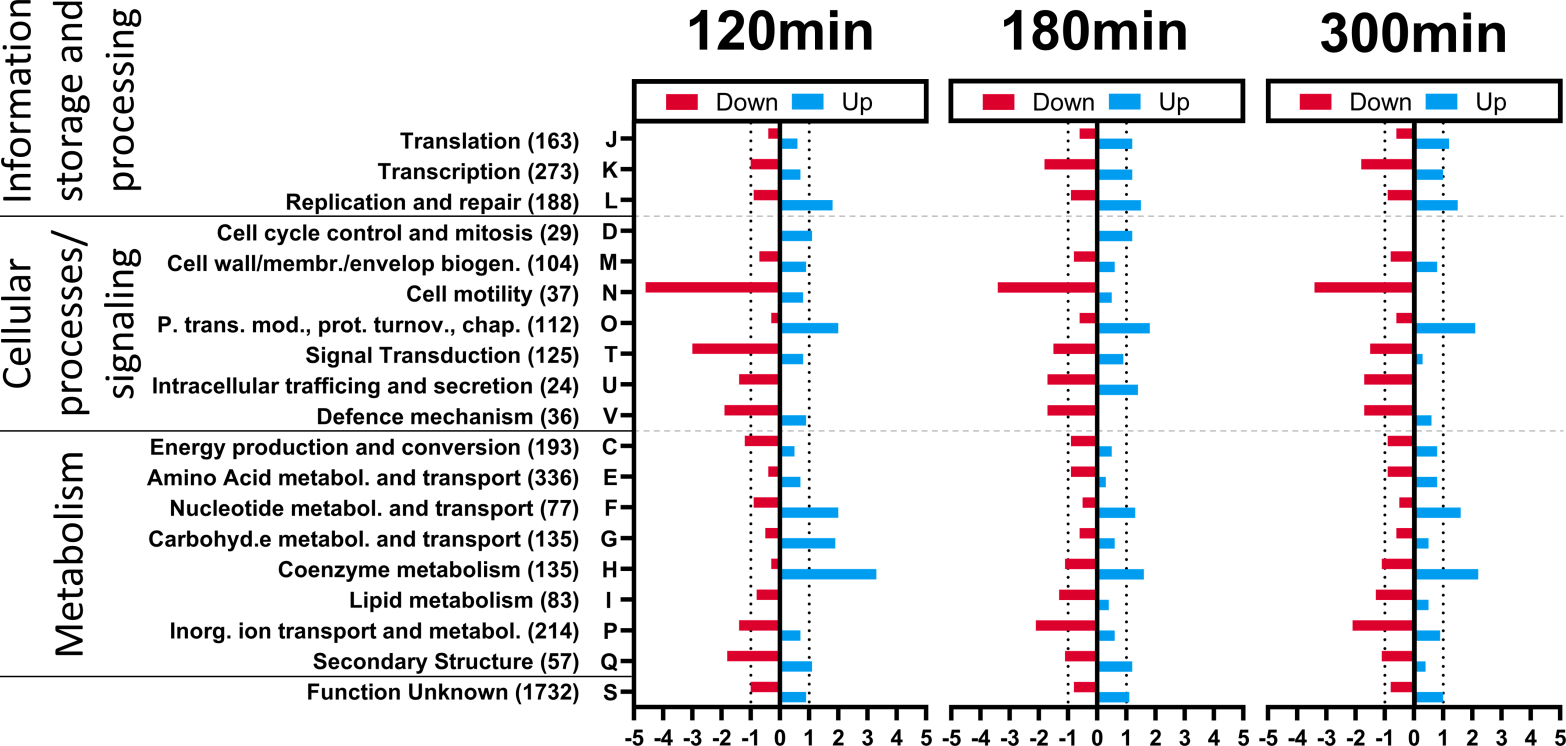
arCOG gene set overrepresentation analysis of differentially expressed downregulated (in orange) and upregulated (in blue) genes of *H. gibbonsii* LR2-5 upon infection of HFTV1 after 120, 180, and 300 min p.i. All genes were categorized in arCOGs and clustered in four groups: information storage and processing, cellular processes and signaling, metabolism, and unknown function. The numbers in parentheses indicate the total number of genes in the arCOGs. Over- or underrepresentation was calculated based on a random distribution of all differentially expressed genes over all arCOGs. For example, in the cell motility arCOG (N), there are 37 genes. The *H. gibbonsii* LR2-5 genome contains 4,053 genes; therefore, 0.91% of all genes belong to arCOG N. At T120, 118 genes are downregulated, of which five belong to arCOG N, which is 4.24%, whereas 0.91% is expected. Therefore, arCOG N is 4.64 times overrepresented of the downregulated genes at T120 [see *x*-axis, left panel, cell motility (37) N]. A value of 1 is expected, and >1 means an overrepresentation, whereas, <−1 indicates underrepresentation of the number of differently expressed genes in that arCOG.

Subsequently, we aimed to identify groups of functionally related genes that are significantly overrepresented among up- or downregulated genes. EggNOGv5.0 was used to classify all differentially expressed *H. gibbonsii* LR2-5 genes in arCOGs. Using the genome of *H. gibbonsii* LR2-5 as a template, 2,321 genes were successfully placed in a functional category, whereas of the remaining 1,731 genes (42.7%), the function was listed as unknown (arCOG category S). All differentially expressed genes, divided into up- and downregulated genes, were linked to their specific arCOG category. The arCOG analysis for the 5, 20, and 60 min p.i. time points was omitted due to the low number of differentially expressed genes, which would distort the analysis (Fig. 5).

### Increased expression of genes involved in nucleotide metabolism

Several genes belonging to ArCOG F, “Nucleotide metabolism and transport,” were strongly upregulated in infected cells, after 120, 180, and 300 min p.i. compared to uninfected controls (Fig. 5). This likely reflects the increased DNA replication and transcription activities in order to replicate the HFTV1 genome. Several genes belonging to this group are among the highest upregulated genes 120, 180, and 300 min p.i. For example, several genes involved in pyrimidine synthesis, such as *dcd* (a dCTP deaminase) (34), *pyrG* (which catalyzes the ATP-dependent amination of UTP to CTP), *guaAb* (which catalyzes the synthesis of GMP from XMP) (35), and *uraA* (a xanthine/uracil permease), are all upregulated fivefold at 120 and 180 min p.i. (see Table 1 for exact fold-change values).

**TABLE 1:** Differential expression of selected nucleotide metabolism genes during HFTV1 infection^*a*^

Gene number	Gene	Fold change	
		T120	T180
HfgLR_03355	*dcd*	5.92	4.10
HfgLR_10300	*pyrG*	5.56	5.22
HfgLR_10295	*guaAb*	4.66	4.40
HfgLR_06265	*uraA1*	3.70	3.23

^*a*^ Fold change values indicate the relative expression levels of each gene at 120 and 180 min post-infection (p.i.) compared with the uninfected control culture.

Dcd in archaea is responsible for the conversion of dCTP to dUTP and thus can help to adjust levels of dCTP in relation to dTTP in the nucleotide pool (36). In the next step, the dUTP can be acted upon by dUTPase to form dUMP, in order to prevent the incorporation of dUMP in the DNA (36). In bacteria, PyrG is a CTP synthetase, which catalyzes the glutamine- and ATP-dependent amination of UTP to form CTP, and its expression is regulated independently of the rest of the *pyr* operon containing pyrimidine biosynthetic genes (35). The role of this gene has not been studied in archaea. The UraA protein involved in uracil/xanthine transport has been described to be highly transcribed in *Ca.* Thalassarchaeaceae*,* but further characterization of this protein in archaea is lacking (37). NrdJ, the adenosylcobalamin (vitamin B12)-dependent ribonucleoside-diphosphate reductase, catalyzes the conversion of ribonucleotides to deoxyribonucleotides, which is essential for *de novo* DNA synthesis (38–40), and this gene is also one of the members of ArCOG F, “Nucleotide metabolism and transport.” NrdJ is, under uninfected control conditions, one of the highest-expressed genes in *H. gibbonsii* LR2-5 and is upregulated 2.0-, 2.1-, and 2.0-fold after 120, 180, and 300 min p.i., respectively, following HFTV1 infection. Like many resources required for the amplification of viral particles, NrdJ most likely is upregulated due to the high demand for nucleotides in order to replicate the viral genome.

### Upregulation of post-translational modification and chaperonins

The arCOG O, “Post-translational modification, protein turnover, chaperone functions,” was also among the enriched arCOGs at 120, 180, and 300 min p.i. Upregulated genes belonging to this class encode several different chaperonins, such as the TCP1 and DNA-J classes. Additionally, a membrane-bound mannosyl transferase, encoded by HfgLR_07500, is significantly upregulated in infected cells. This protein is likely involved in the addition of N-linked glycans, which are required for N-glycosylation of archaeal surface proteins (41). Upregulation of this class of proteins (arCOG O) could be explained by the high demand of protein expression, folding, and post-translational modification machinery due to the massive production of HFTV1 proteins in the hours following infection, leading up to the assembly of mature viral particles.

### Upregulation of the coenzyme metabolism by the precorrin pathway

**Fig 6:**
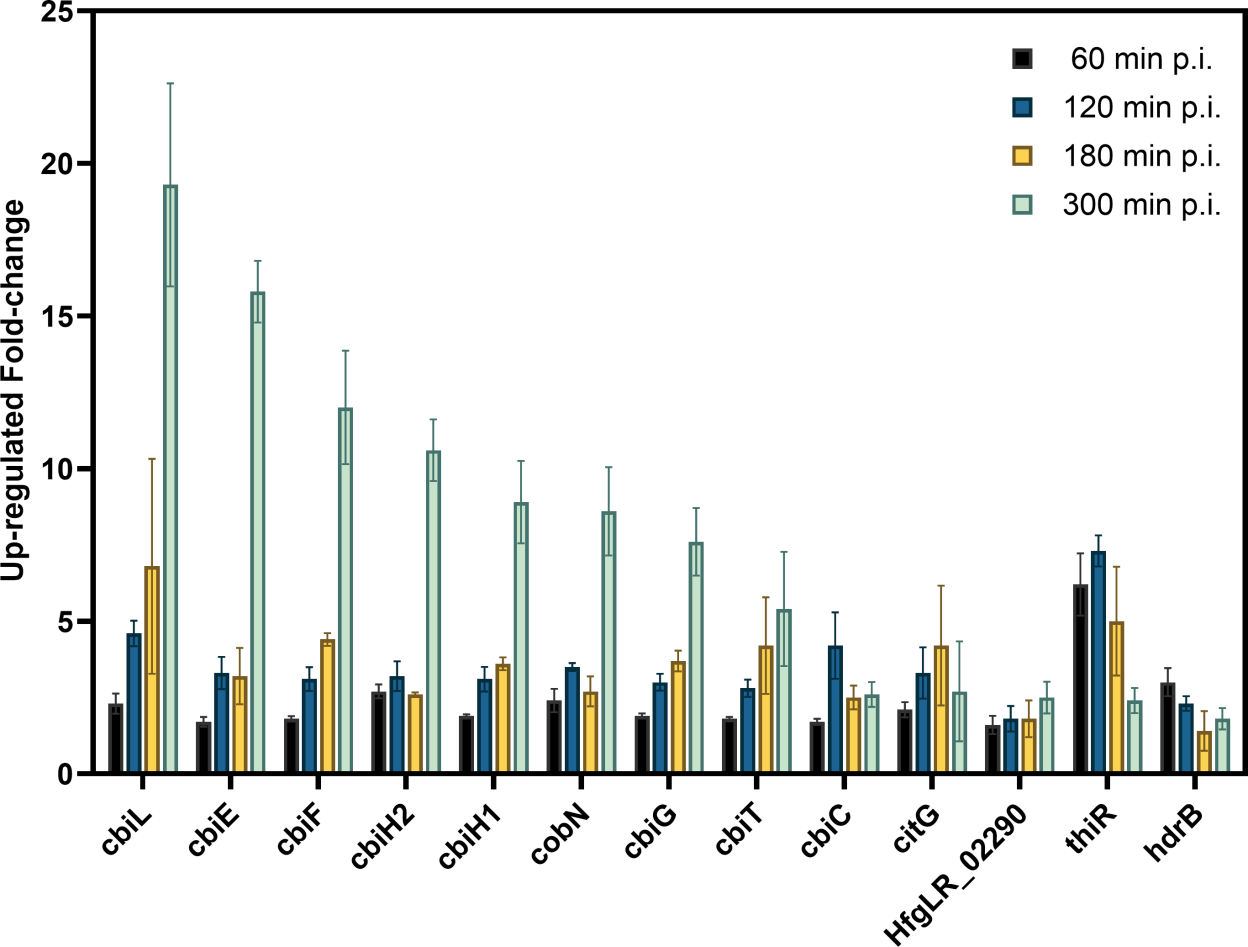
Fold change of the major upregulated genes of the “Coenzyme metabolism” arCOG H at (from left in each group of bars) 60, 120, 180, and 300 min p.i. Error bars indicate the standard deviation of three biological duplicates.

At 120, 180, and 300 min p.i., among all upregulated genes, the “coenzyme metabolism” arCOG (H) was 2.4-, 3.3-, and 1.6-fold overrepresented, respectively (Fig. 5). ArCOG H contains (in total) 135 genes, of which 13 are significantly upregulated at 300 min p.i. (DESeq2 analysis; *P*-value < 0.05) (Fig. 6). Ten out of the 13 upregulated arCOG H genes (annotated as cbi and cobN) belong to the precorrin pathway, which produces precorrin-2, the precursor of cobalamin (or vitamin B12).

In bacteria and archaea, vitamin B12 is required by various enzymes, including methionine synthase (42), ribonucleotide reductase (38–40), glutamate and methylmalonyl-CoA mutases (43), ethanolamine ammonia-lyase (44), and diol dehydratase (45). We hypothesize that the upregulation of the precorrin pathway reflects a demand-driven need for vitamin B12, which is required for, e.g., NrdJ, the vitamin B12-dependent ribonucleoside-diphosphate reductase (see above [40]).

### Cell motility genes are downregulated

Among the downregulated genes, the arCOG category N, “cell motility,” was significantly overrepresented, with 4.6-, 3.4-, and 3.4-fold more representatives than expected at 120, 180, and 300 min p.i, respectively (Fig. 5). Belonging to this arCOG “cell motility” are genes encoding adhesive pilins. Specifically, *pilB4* and *pilC4* showed significantly reduced expression in infected cells. PilB and PilC are an ATPase and membrane anchor, respectively, that make up the motor of adhesive type IV pili in archaea (46). The pilins that make up the filament are encoded by *pilA* and loaded into the filament by the PilB/C complex. These pili are homologous to bacterial type IV pili and are involved in adhesion and biofilm formation (46). Haloarchaeal genomes usually contain multiple different copies of *pilA*, *pilB*, and *pilC*; for instance, the *H. volcanii* genome encodes five PilB/PilC pairs.

*H. gibbonsii* LR2-5 encodes four PilB-PilC pairs and six different PilAs. All of these remain stable throughout the infection, with the exception of PilB4-PilC4, which is homologous to the same pair from *H. volcanii*. The expression of the PilB4-PilC4 pair is under normal uninfected conditions higher than those of PilB3-PilC3. Downregulation of PilB4-PilC4 leads to levels that are only lower than that of PilB3-PilC3. Thus, downregulation of PilB4-PilC4 could result in fewer piliated cells or in cells with pili that have a different PilA composition. As the pili from *H. gibbonsii* were not yet analyzed in detail, the impact of PilB4-PilC4 remains to be established.

**Fig 7:**
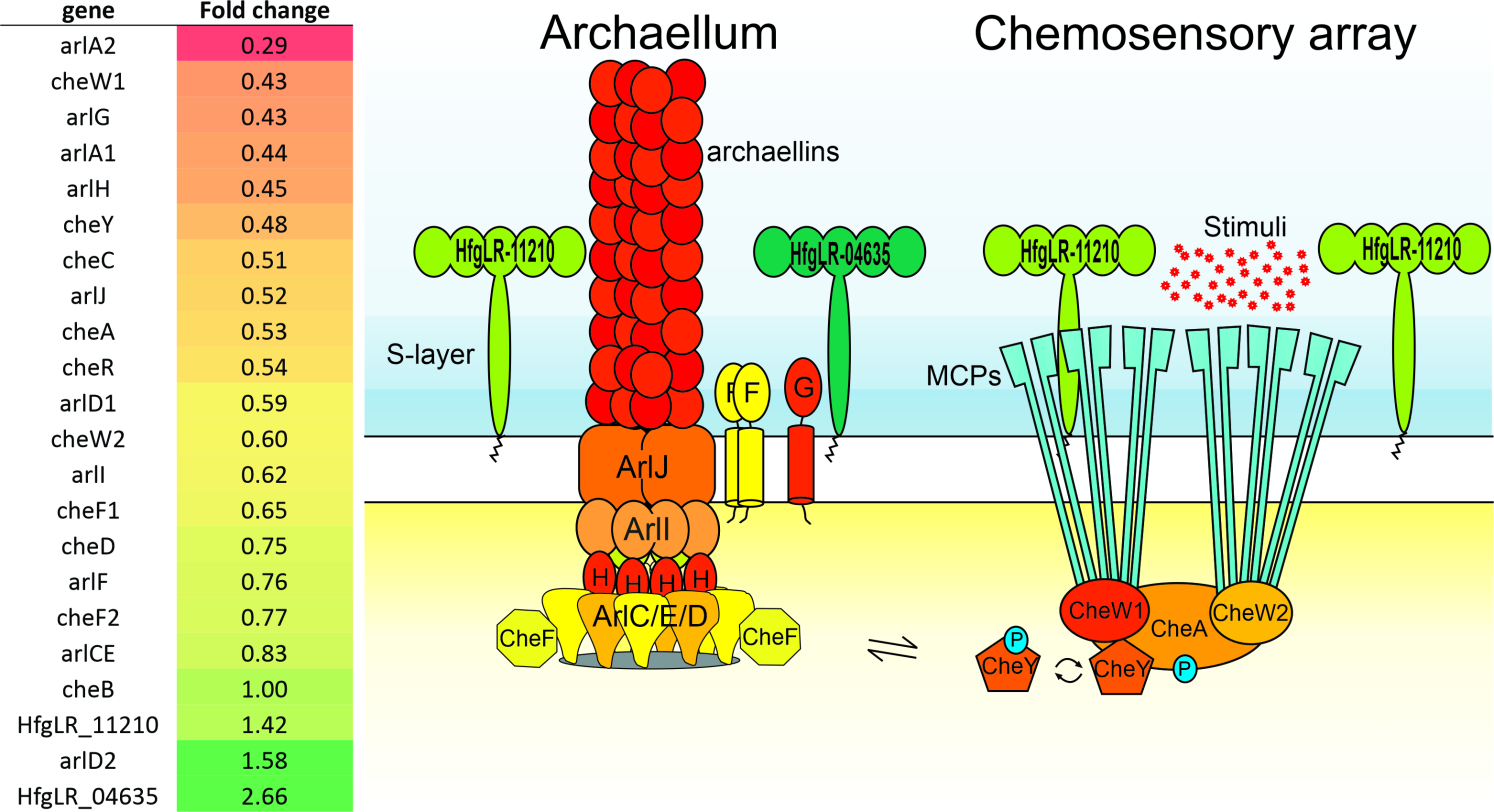
Fold change values of a subset of genes of the archaellum and chemosensory array of the “cell motility” arCOG N at 120 minp.i. in which the colors used in the image match the color scheme of the fold change values indicated on the left. HfgLR-11210 and HfgLR-04635 are the S-layer encoding genes.

Five significantly downregulated genes that are part of the “cell motility” category—*arlA1*, *arlA2*, *pilB4*, *pilC4*, and *cheW1*—showed log_2_ fold change values of −1.44, −1.79, −1.39, −1.21, and –0.93 at 120 min p.i., respectively. ArlA1 and A2 are the main archaellum components of *H. gibbonsii* that form the filament of this rotating motility structure (7). Besides the downregulation of the archaellins *arlA1* and *arlA2*, almost all genes of this archaellum operon were downregulated starting at 120 min p.i. (Fig. 7). The other arl genes in the operon*—arlG*, *arlJ*, *arlF*, and *arlCE*—encoding different components of the archaellum motor were downregulated with log_2_ fold change values of –1.21, −0.94, –0.39, and –0.26, respectively. Thus, HFTV1 infection leads to clear and strong downregulation of the archaellum machinery.

CheW1 is a component of the chemosensory arrays, together with CheA, the response regulator CheY, and the MCPs (methyl-accepting chemotaxis proteins). The latter detect extracellular signals and transfer them via the chemosensory arrays to the archaellum, enabling them to respond adequately to environmental cues. Besides *cheW1*, also *cheC*, *cheR*, *cheW2*, and *cheD*, all encoding components of the chemotaxis system, were downregulated 1.9-, 1.8-, 1.7-, and 1.3-fold, respectively, at 120 min p.i. Their expression decreased even further at 180 and 300 min p.i. (Fig. 7). Another gene in the top 10 of downregulated genes at 120 min p.i. is *hfgLR_13260*, which is annotated as a PAS domain-containing histidine kinase. PAS domain-containing proteins are known to sense environmental factors, such as light, nitrogen, oxygen, or redox potential. These sensing proteins are usually signaling downstream systems, such as the motility machinery. For example, the methyl-accepting chemotaxis proteins, which are the sensory receptors of the chemotaxis system (see below), can contain such PAS domains. It remains to be established whether *hfgLR_13260* transfers signals to the motility machinery.

Together, these results suggest that the expression of the large part of the chemotaxis machinery is downregulated after HFTV1 infection. Reduced expression of both the archaellum and the chemotaxis system suggests that HFTV1-infected cells become less motile after infection.

### Cells show reduced swimming behavior after infection

**Fig 8:**
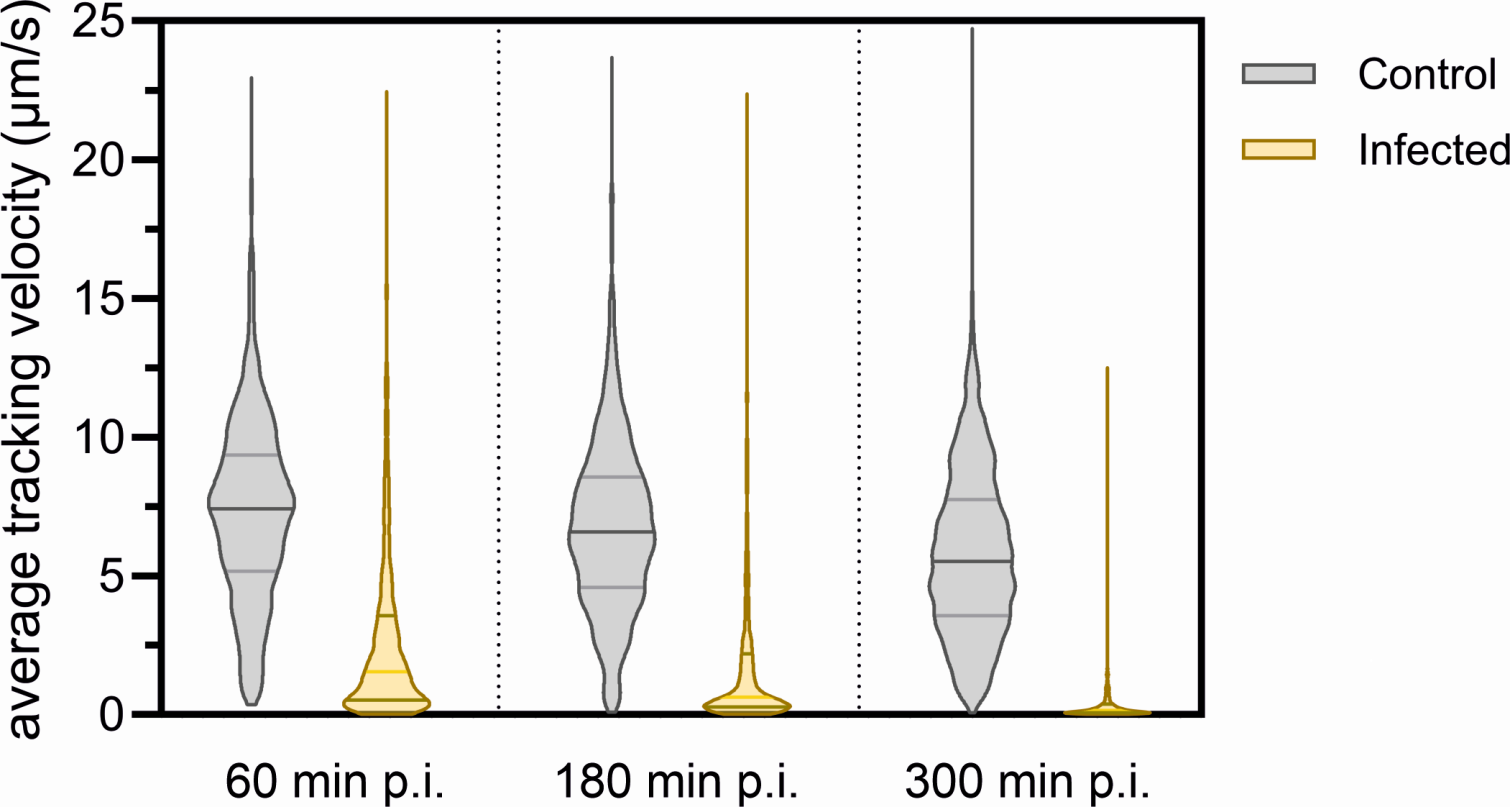
Infection of HFTV1 inhibits swimming motility of *H. gibbonsii*. Violin plots depict the distribution of mean tracking velocities (µm/s) for cells in control (gray) and HFTV1-infected (yellow) cultures at 60, 180, and 300 min p.i. Tracking and quantification were performed using TrackMate. Average tracking velocity was measured over 15 seconds for each indicated condition and time point. HFTV1-infected cells exhibited a reduced mean tracking velocity compared to uninfected controls throughout the infection cycle, indicating virus-induced impairment of cellular motility. Sample sizes were as follows: 60 min p.i., *n* = 1296; 180 min p.i., *n* = 973; and 300 min p.i., *n* = 1705. Statistical analysis was conducted using unpaired Student’s *t*-tests comparing infected and control groups at each time point. All comparisons yielded highly significant differences (*P* < 0.0001).

Time-lapse microscopy was applied to analyze whether the downregulation of the archaellum and chemotaxis operons leads to obvious reduction of swimming behavior of infected cells. Cells were grown to mid-exponential phase, infected with an M.O.I. of 10, and compared with an uninfected control strain grown under similar conditions at different time points p.i. Cells were visualized by time-lapse microscopy under native growth temperature. Time-lapse movies were made, and cells were tracked (Movies S1 and S2). The average velocity of cells was measured in each field of view (Fig. 8). HFTV1-infected cells showed a greatly reduced tracking velocity compared to healthy control cells. While control cells swim with an average velocity of 5.4 µm/s, 1 hour after HFTV1 infection, the velocity was already reduced to 2.2 µm/s and further decreased to 0.4 µm/s at 300 min p.i.

Thus, HFTV1-infected *H. gibbonsii* LR2-5 cells are significantly less motile compared to non-infected controls. The reduced motility likely results from downregulation of the archaellum operon and may reflect either a host stress response to viral infection or a redistribution of energy resources required for viral replication and production. While it remains possible that rendering their hosts non-motile could provide an advantage for the virus—for example, by promoting a sessile lifestyle in biofilms where the availability of potential new hosts is higher—this remains speculative and would require further investigation to establish a direct causal mechanism.

Inhibition of expression of motility genes has been reported to occur after infection by several bacteriophages. For example, motility genes are repressed early in the infection cycle of bacteriophage PaP3, infecting *Pseudomonas aeruginosa*; the filamentous SW1 phage, infecting the bathypelagic bacterium *Shewanella piezotolerans*; and the filamentous ϕAFP1 phage, infecting the Gram-negative marine bacterium *Alteromonas abrolhosensis* (47–50). It is generally assumed that these viruses inhibit the expression of motility genes in order to balance the high energy demand associated with viral replication and particle production. This mechanism is likely also occurring after HFTV1 infection, together with the manipulation of host metabolism and chaperonin expression observed in this study. However, further work will be needed to clarify whether the downregulation of motility genes is a targeted viral strategy or a more general stress response of the host. Virus-beneficial changes in the host transcriptional landscape during infection have also recently been observed for halovirus His2 infecting *Haloarcula hispanica* (51), as well as in some, but not all, viruses infecting hyperthermophilic archaea, and thus appear to be an important aspect in the reproductive strategy of archaeal viruses (28, 52–54).

### Conclusion

In conclusion, the data presented provide a detailed picture of transcriptional regulation during the infective life cycle of the model archaeal virus HFTV1. We show that HFTV1 infection significantly impacts host gene expression. The observed increased expression of genes involved in nucleotide metabolism and chaperonins is likely orchestrated to support viral replication and assembly of viral particles. We also observed a strong upregulation of the precorrin pathway responsible for vitamin B12 production, which is needed for B12-dependent enzymes. Lastly, upon infection, motility and chemotaxis genes are rapidly downregulated, corresponding to the reduced swimming speeds of infected cells observed by time-lapse microscopy. Cellular motility could be actively repressed by the virus to redirect resources from energy-costly motility toward other processes, or it might be linked with specific benefits for viruses in sessile cells, such as a potentially reduced likelihood for subsequent encounters with viruses. Our analysis of viral transcripts, on the other hand, shows that HFVT1 expression is tightly regulated throughout the infective life cycle, similar to what is typically observed for bacterial viruses. Interestingly, though, we further observed fine-tuned temporal transcriptional initiation from within gene clusters, which can provide HFTV1 with extra opportunities for individual gene regulation.

Additionally, the high number of transcripts vs. the number of predicted genes in HFTV1 is in stark contrast to bacterial viruses, where generally many genes are arranged in a few long transcripts. Another feature in the HFTV1 transcriptional landscape is the prominent role of asRNA potentially involved in the late regulation of expressed genes. The observed important role of asRNA in this archaeal viral model might be linked to the high presence of asRNA in archaea compared to bacteria (18).

Generally, in comparison to temporal transcriptomic analyses that have been performed on other archaeal viruses, the complexity of the large number of differentially regulated sense and antisense transcripts in HFTV1 appears to stand out. However, to date, almost all other archaeal viruses for which time-resolved transcriptomic data exists are hyperthermophiles and/or viruses with a significantly smaller genome (<20 kb) (51, 52, 54–56). The only other halovirus for which time-resolved microarray data has been published is the chronic virus His2, which, with a genome of 16 kb encoding 35 ORFs, shows clear temporal regulation but only has three distinctly regulated regions (i.e., early, middle, and late [51]).

This work provides unprecedented insight into transcriptional regulation of haloarchaeal viruses, which will be valuable for future studies of archaeal transcription and increases our insight into the mechanisms by which archaeal viruses take control of their host cells.

## MATERIALS AND METHODS

### Growth and infection

Growth and infection procedures were carried out as previously described (12), with detailed conditions provided in the Supplemental text.

### Probe design, synthesis, and chemical labeling

Polynucleotide probe design, synthesis, labeling, and specificity testing were performed according to established protocols (57), with probe sequences and labeling details listed in Table S1 and Supplemental text.

### Virus targeting direct-geneFISH (virusFISH)

VirusFISH was conducted following the direct-geneFISH protocol by J. Barrero-Canosa and C. Moraru (58), with modifications described in the Supplemental text.

### Bioinformatic genome analysis

Genome annotation and analysis were conducted using Pharokka and associated tools, as described in Supplemental text.

### Sample preparation and RNA isolation

Flash-frozen cell pellets were thawed on ice, and total RNA was extracted using RNeasy Plus Mini Kit and RNeasy MinElute Cleanup Kit (Quiagen, Hilden, Germany) according to the manufacturer’s instructions, following protocol 1 for purification of total RNA containing miRNA. RNA concentrations were measured by spectrophotometer using a Nanodrop (Implen, Munich, Germany).

### Expression analysis

RNA-seq and differential expression analyses were performed by Vertis Biotechnologie AG and analyzed using standard pipelines (BowTie2, DESeq2, SeqMonk), as described in Supplemental text.

### Swimming analysis with time-lapse microscopy

Swimming behavior was assessed using time-lapse phase-contrast microscopy at 45°C, as detailed in the Supplemental text.

## Data Availability

Raw and processed transcriptomic data are available without restriction at Zenodo and can be accessed via the following DOI: 10.5281/zenodo.15221547. Any code used for data processing and appropriate documentation can be found in Materials and Methods. Please note that the genome annotation provided in this study is preliminary and based solely on expression profiling and homology predictions, without experimental validation. These annotations should therefore be considered provisional.

## AFTER EPUB

*[This article was published on 22 September 2025 with an error in Fig. 3. The figure was corrected in the current version, posted on 10 October 2025.]*

## SUPPLEMENTAL MATERIAL

Supplemental Material msystems.00704-25-s0001.pdf Supplemental text, tables, figures, and movie legends.

Movie S1 msystems.00704-25-s0002.avi Time-lapse light microscopy of *H. gibbonsii* cells under standard conditions.

Movie S2 msystems.00704-25-s0003.avi Time-lapse light microscopy of *H. gibbonsii* after HFTV1 infection.

ASM does not own the copyrights to Supplemental Material that may be linked to, or accessed through, an article. The authors have granted ASM a non-exclusive, world-wide license to publish the Supplemental Material files. Please contact the corresponding author directly for reuse.
