# Supplementary material for: The infection cycle of the haloarchaeal virus HFTV1 is tightly regulated and strongly inhibits motility of its host
Source: mSystems. 2025 Sep 22;10(10):e00704-25. doi: 10.1128/msystems.00704-25 (PMC12542753; doi:10.1128/msystems.00704-25)
Supplement: Supplemental Material — Supplemental text, tables, figures, and movie legends. [file msystems.00704-25-s0001.pdf]

**Supplemental Information**

# SUPPLEMENTARY TEXT

## Growth and infection

*Haloferax gibbonsii* LR2-5 was grown aerobically in liquid modified growth medium (MGM) at 37 °C as described by S. Schwarzer et al. (1). This medium contains 18% artificial salt water (SW) and yeast extract (0.1% w/v Oxoid) and peptone (0.5% w/v Oxoid) (2). *H. gibbonsii* LR2-5 was grown in liquid medium until $OD_{600nm}$ ~1. Then the culture was diluted and divided over 6 ×100 mL Erlenmeyers with MGM medium to a theoretical $OD_{600nm}$ of 0,05. After 24 hours of growth, when the $OD_{600nm}$ was ~0.5, to 3 of the 6 Erlenmeyer, HFTV1 particles were added to a multiplicity of infection (M.O.I.) of 10. HFTV1 stocks were prepared as described in Schwarzer et al 2023 (1). The cultures were kept at 37 °C and incubated aerobically. Samples for RNA extraction were taken at 5, 20, 60, 120, 180 and 300 minutes post infection (p.i.) from infected and non-infected cultures in three biological replicates. At each time point, 2 mL samples were collected and immediately centrifuged at 3000 × g for 2 min. The supernatant was discarded and the cell pellet was flash frozen in liquid nitrogen and stored at -80°C.

## Probe design, synthesis and chemical labeling

A total of 57 polynucleotide probes targeting the 38 kb viral genome of HFTV1 (NCBI accession no. NC_062739.1) were designed using the genePROBER Software (gene-prober.icbm.de/) (3). Sequences of the 300-bp polynucleotides are listed in Supplemental Table 1. Selected probes were chemically synthesized as gBlocks® gene fragments (500 ng per polynucleotide) by IDT (Integrated DNA Technologies, San Jose, CA, USA), resuspended in 5 mM Tris, 1 mM EDTA, pH 8.0 prior to labeling with the ULYSIS™ Alexa Fluor™ 594 Nucleic Acid Labelling Kit (Thermo Fisher Scientific, Waltham, MA, USA) according to the

manufacturer's instructions. 2 µg HFTV1 targeting polynucleotides were labeled in a single reaction and subsequently purified using Micro Bio-Spin® Columns with Bio-Gel® P-30 (Bio-Rad Laboratories Inc., Hercules, CA, USA). Labeling efficiency was determined by spectrophotometric measurement using a NanoPhotometer® N50 (Implen, Munich, Germany) and calculated 9.12%. Probe specificity was checked against uninfected cells to exclude the possibility of nonspecific binding to cell structures.

**Virus targeting direct-geneFISH (virusFISH)**

Fluorescence in situ hybridization (FISH) was performed in accordance with the direct-geneFISH protocol of J. Barrero-Canosa and C. Moraru (4) . Virus infections were performed as described in growth and infection using an M.O.I.. of 10. Cells were fixed in a paraformaldehyde solution (PFA) (Thermo Fisher GmbH, Kandel, Germany) as described in the “Core” direct-geneFISH protocol. 10 µl of fixed cells were spotted within a Silicone IsolatorTM (Grace Bio-labs, Bend, OR, USA) on a SuperfrostTM plus adhesion microscopy slides (Epredia Netherlands B.V, Essendonk, The Netherlands), air-dried and dehydrated by washing in ethanol with 50, 70 and 100% (v/v) for 10 minutes at room temperature. Haloarchaea have an S-layer consisting mainly of glycoproteins and no peptidoglycan, which makes them naturally more permeable and does not require an additional step to permeabilize. The samples were overlayed with a solution of 80 µl hybridization buffer containing 35 % (v/v) Formamide, 5 × SSC buffer (saline sodium citrate, pH 7.0), 20% (w/v) dextran sulfate, 20 mM EDTA, 0.25 mg $mL^{-1}$ sheared salmon sperm DNA, 0.25 mg $mL^{-1}$ yeast RNA, 1 × blocking reagent, 0.1% (v/v) sodium dodecyl sulfate, nuclease-free water, and probes were added at a final concentration of 30 pg $µl^{-1}$ for each polynucleotide probe. To denature probes and templates, the samples were incubated at 92 °C for 40 min, followed by 2 h hybridization in a humidity chamber at 46 °C. Following hybridization, samples were rinsed in wash buffer I (2x

SSC, 0.1% SDS) for 5 minutes at 48 °C, and then in wash buffer II (0.1x SSC, 0.1% SDS) for 30 minutes at 48°C. Finally, the samples were washed for 25 min in 1 × PBS and 1 min in ultra-pure water and air-dried. Cells were counterstained for 10 minutes in the dark with 10 µl of a mixture of SlowFade™ Diamond Antifade Mountant with 4′,6-diamidin-2-phenylindole (DAPI) (Thermo Fisher Scientific, Waltham, MA, USA) and covered with a #1.5 high-precision coverslip (Paul Marienfeld GmbH, Lauda-Königshofen, Germany). Images were acquired with a 100 × Plan-Apochromat oil objective (Numerical Aperture, NA = 1.46) on a Zeiss Axio Observer 7 inverted fluorescence microscope (Carl Zeiss Microscopy GmbH, Jena, Germany) equipped with a Colibri 7 LED illumination system and a Prime BSI Express sCMOS camera (Teledyne Photometrics, Tucson, AZ, USA). Phase contrast was imaged with an exposure time of 20 ms. DAPI was excited using an LED at 385 nm, and emission was collected using a BP 460/50 nm filter with an exposure time of 20 or 70 ms. Alexa Fluor 594 was excited using an LED at 555 nm, and fluorescence was detected using a BP 630/75 nm emission filter with an exposure time of 20 ms. Image acquisition and processing were performed using Zeiss ZEN (Blue Edition) software (version 3.5).

**Bioinformatic genome analysis**

Pharokka v 1.7.5 (5) was used to update the annotation of the HFTV1 reference genome (NC_062739.1). More specifically PHANOTATE (6) was used to predict coding sequences (CDS), while tRNAscan-SE 2.0 (7) was used to predict tRNAs. The respective functional annotations were assigned by matching each CDS to the PHROGs (8) VFDB (9) and CARD (10) databases using MMseqs2 (11) and PyHMMER (https://github.com/althonos/pyrodigal-gv). By using Mash (12) contigs were matched to their closest hit in the INPHARED database (13). The functional annotation was refined using Phold (https://github.com/gbouras13/phold) which uses ProstT5 (14) for the generation of protein structures for each CDS. Followed by a

comparison against a database of predicted viral protein structures using FoldSeek (15). The resulting output plots were created using pyCirclize (https://github.com/moshi4/pyCirclize).

**Sample preparation and RNA isolation**

Flash frozen cell pellets were thawed on ice and total RNA was extracted using RNeasy® Plus Mini Kit and RNeasy MinElute® Cleanup Kit (Quiagen, Hilden, Germany) according to the manufacturer's instructions, following protocol 1 for purification of total RNA containing miRNA. RNA concentration were measured by spectrophotometer using a Nanodrop (Implen, Munich, Germany).

**Expression analysis**

The RNA sequencing, including the rRNA depletion and cDNA synthesis, was performed by Vertis Biotechnologie AG (Freising, Germany). For sequencing, individually barcoded cDNA libraries from all samples were pooled and sequenced together on an Illumina 500 system using 1×75 bp read length. On average, 10.5 million reads per sample were obtained. The FastQ files were run through a FastQC (https://github.com/s-andrews/FastQC), BowTie2 (https://github.com/BenLangmead/bowtie2), SamTools (https://github.com/samtools/samtools) pipeline and the resulting BAM files were analysed using SeqMonk (16) V1.48.1 in which the *H. gibbonsii* LR2-5 strain (Genome assembly ASM1496974v1) or Haloferax tailed virus 1 (HFTV1, Genome assembly ASM420877v1) were used as reference genomes. All *H. gibbonsii* LR2-5 genes were analysed, in an intensity difference statistical test (DESeq2 in SeqMonk) in which a statistical difference of below 0.05 was used ($p<0.05$). The EggNOGv5.0 (http://eggnog-mapper.embl.de) was subsequently used to functionally classify all *H. gibbonsii* LR2-5 genes in the various archaeal clusters of orthologous genes (arCOGs).

Relative coverage was calculated for genes as ($RPKM_{gpXX}$ at $t_n$/max[$RPKM_{gpXX}$])*100% and for TSS as ($CPF_{TSSXX}$ at $t_n$/max[$CPF_{TSSXX}$])*100%, with CPF refereeing as read counts per feature (i.e. number of reads mapping to a single nt within the TSS). TSS were determined highlighting regions for which the following calculation gave values of 200 or greater; i.e. a coverage increase of 200 within 3 nts: [coverage at position X] - [coverage at position X±3nt]. Next, the read mapping was manually inspected and the TSS annotation was visually confirmed.

**Swimming analysis with time-lapse microscopy**

For analysis of swimming behaviour, an overnight culture was diluted in MGM medium, to a theoretical $OD_{600nm}$ of 0,05. After 24 hours, when the $OD_{600nm}$ was ~0,5, the culture was infected with HFTV1 to a M.O.I. of 10. Samples were taken at different time points post infection. In all, 1 mL of each culture was placed in a round DF 0.17 216 mm microscopy dish (Bioptechs, Butler, PA, USA) and observed with an 40 × Plan-Apochromat objective (Numerical Aperture, NA = 0.95) in the PH3 mode with a Zeiss Axio Observer 7 (Carl Zeiss Microscopy GmbH, Jena, Germany) equipped with a heated XL Multi S1 Incubation chamber (PeCon GmbH, Erbach, Germany) combined with the TempModule S1 temperature control unit (PeCon GmbH, Erbach, Germany), heated to 45 °C running Zen 3.5 software.

## SUPPLEMENTARY DATA

### Supplemental Tables

**Supplemental Table S1:** Polynucleotides used for virus targeting direct-geneFISH with HFTV1.

| No. | GC content (%) | Sequence (5' - 3') |
|---|---|---|
| 1 | 56.3 | cgactcgtggggcgagacagagacgggcgtggaagccaccgagatcacggtctcgtctgacgtggtggaagtgacggaagcagacttacaacacgactaaccaccacggcttactacacgcatgagtacacagtcgttcaatttcacatggaaactctcagccaaacagcgggagttattcgagtcagacgcgcggttccgcgtgggcatgatgggtcgccggttcgggaagaacgaagtctcgacggcgatagaagtcgattacgccacccaaccccacaaatacacgttcggcacaga |
| 2 | 56.0 | tgacccagacggcggtcttgtgtggcatatcgccccgacctatcggcaggcgtatcgacatggctatctcaaagtcatggagaaactgccggatgcgttagtagacgatgataacacccgtgggagtgagtggtcgccgtccaaagtgacgcttgtgacgggtttcgagttagagttcctttcctacggcaaccccaagggcttgcaggggggagggcgtggacttgattgttggcgacgagtgggcctattcggaccccgagatatgggacaaagacttgcgtccgatgctcttagatac |
| 3 | 51.7 | ccgagagtgcgaccgaacctttgagtttgacgacgatgatacgcttgatgatgccgctgcggtgtggaacaaccacgcggtgtggaacaaccacgtaatgagcactccacagtaacggcacttctccggctaagagtctttatctggaatagtgcagttacccaaagtcaccaacagtaactattaaggcagagcgtttctactaccaatacgtgacgaaagcagtcaaagtaccgtcgccagtgtacgaccgagtgacacgagaagcagagcgcaaagacgtaccgcgcggcgttatca |
| 4 | 51.7 | gtcgagtacacgaacgcgaaaaacgaggtgcatgcatgaacatccatactcgttgcccggcgtgtgaccaacatggggttgaagtcaccgcccaagacaaagaggcgttagtctgcatgaacgcgggttgtcgggtattcgtgtttgcaacgtatctcgggtgatagagtgacggactatctattgctcaggggtgcgtgtacccaactcatatttgaggaggaaaaggccttagaaaacgagtggcacacgggcgacgaatacgacaatcggctatggaaaatcgaacgtctcaaacgg |
| 5 | 53.3 | tttaagaaatacgttgaactccaccgagagtttaacgaatagccgaaacccttagtccctgcccttaatacggggaagtatgaacgtctcacccgccgaacgccttttactcgctgtaacgctccttgccgtcttgtatcgcatcgcccgcgaagagtggccggttggccttgaagtcgaatccacggagtacaactaatgccgaagtacaaccttcgcattggcaatcgccgtgtcccaatcgcatcaaccgacacgccactctccgaagcaatcgggaaacgccttgcatccagcacg |
| 6 | 52.0 | cactcttttcagagtttgacaatatcccgagttcacggcttatggagttgcgtcagacctttgctgacgaacttggtacggacaacttcacgcttgactctatcacggacgcactcatggactttgaggcagaccttactcgggatgccgcagagcgtatcgctcgcacagaatcatcggcagtattgaaccatgcaagggagatttcttacgaggagcgtggggagggtaacgaactcttctattggacaggcgcggacttaggagattcacgccagaccgaagcgtgtgcgtggttaa |
| 7 | 55.0 | tccgtcagacaaacccccttctcaggaggtacgcccgtgccgatgaacgaacttcgggatatggtcgacgaagcaccgtcacatgatgatagcatggacaacaatctcgcacggcctgattcgtgggttgtgcaccccaacgagagaagttcgttcgtgaaggcacccccgaactgggaacaactatagcccaaacgcttactctcttgccgtggacgtaggagtatgagcgacttgaaaggcgcactcacagacatcgacggcgttggcgacaagacggcagacaagattctcaacgtgg |
| 8 | 56.3 | cagaccccgacgaagtgcgggaaacgctcgatatgttcgggtttgacggccttgacgagatgaccgacgacgaagtgacggacatggccgaagacctgcacgaagaccttacgtccgaccttgaaggcatggacgaagaaggcgagggcgaagaaatgggcgaaggcgaagaatacgacgacgacgaagaagaagacgaacaggagat |

| | | |
|---|---|---|
| | | gcaagaaggcgaggacatggaagcactgaaagagcaagtgtcgtcgctttcgtcccggcttgaagacctcgaagacgccatgagtcaggcca |
| 9 | 55.0 | tgagtgccgacgaagtgaccgaagaactccaagaggcgaaagaagaactcgcggcggcagagacggtggccgaactcaccgaggcgaaagaggaactggataagcgtctgtcggaactcgaaagtcagggcaagacgccgaagacgctttcggatgcgaacaccgatgccgaagagtgggaacccacgtatgactcttctccggtatcgccgtctgggtggtaggtcactcagaattgactttccgtaggcccttactttttacgtagcactgtatatccgtagccagaagcatggctac |
| 10 | 52.7 | agtctgtaaatgttgaaactccgaacgggttagactcgccaagtcgccccgagttagaatttaactggtatgccgttggccgcgcagaggtgtctattgactacaccgacgacaataaacagaaaacgaccacgccagcaacactcacagttgatgacgattggttaagcgacgacccaactggccgggttggattccgtctggatgtgtcgaacggcggtatccaactcgaagcggggagcattgcgtacattgtgaaaagtggaacgccgccgacttctcgggcaaagcctcacgtat |
| 11 | 52.0 | tcagttcaagcgaactgaatcagattccggccgatgggtatggtgtcgttggggcattccgaattgacccgaatcgtgataacgtgttcaccacgattaacaacgttgtcgtaacttccgagcaaagcgttgacgttgaactcaacttgaaagctgttccagcgagcgcaacagatgcggatttcttggacccagacgacgacggcacggacgaaggtccggcatatccgcggagtaatagcccacaaaattcagtgattcaatggaccccgaacgtctcaacgttcccgacacgcacct |
| 12 | 54.3 | atgccgtagatggaagtacgattccgaacgggcgttctgtcggtgcggctgttgaaaccagtgcgggggtcggagcagggacacaaaagacgggggaaacgtttgttcaaaagcgacccatctatcccgacgacgttgtgttgatgataggtaacaccccagacgcaagcactgccgcggatgtggatatcatcctcgggagcgaccaagactggtaagtccaccgataaacctttgccaccacgtatcaatatggtagaatgtaatgcaactacgacgctcgccggggatgcgtccgat |
| 13 | 54.7 | ggaccgggactggcatcaggagagggcgagggcgcgagagcaagcgtatagcagtgacttaacctcgcaattctccgagtcggagattgtcaagtacgagcttgacacggcacagattgacggaagtgacaaccccggacgtacatctggaaccggacgattgacctcttcgggatgaacggcacggacgtgcgggagttgagaaaccgatgattcacgtctctgttgaccacctccaagcggacaacgccgaaaagttcagagatgcaatcaagagtgtgacagacgaagaagtgatt |
| 14 | 53.7 | aggagttcgtcgacgactcgtatagcgcggcagagttagaccggatggacgggcgagagttgcagagtctcgcggccaaacacccgactgacgaagtaaacggcaagagcaaggccgtggatattcgggacgcactcgaaggcaaaacacgggttagtgtggaatgacagaagacaagcggccatacatttacacgaactttcagacgacgcacggaagcgttgagttgacggttgaaggttcggagggtgaaagcacgtcagacatagagggcgtgtttgacgacaaacttgagaaagt |
| 15 | 55.3 | ggttgacgcccagacgcgacttgatgaggacgatgacgaaaagcgaggtgtgcaatgatttgcttcgtctgccaacaaccccgcgagtgtgacgagacagtgaaaggcgaaccgtggtgtgggtgtgaactatgagtgggtggaaatgttcaaacctcgcgtgccaacgtccacttaatgcacgtattcaaggaacgtcagggtgggcgttttgcggggagtgcgcggcgagtgacgaactaacagtcaccggggacttggtgttgatggaagaatgagtgcgcatcgcccaaccatgtc |
| 16 | 51.3 | tttgcaactcgttcaagagacggcaatcgagaggggcgagacagacgagatgattttcactcgggaggagttcgcacagtttgaccaacacttcaagcgtcgtctggcaggtgccgcgaataccgatgagatttctggtaggtcgacactgctggaaataaaatcgtacctcgtttgtcagtatacgcttgatgagttcgtggattcggacgagtgaccacacaactatgccacgccacaccaatcactaaccatgccggacccatctatcgacgaagtgagtaagagcgattgggacgc |
| 17 | 55.3 | actgacgacacaagagcaagacgacattatctcgcaagtcgagaacctgagttccacggggtgggtaaacacgtcacgagagcgcaaggccgaagcaatcagaagcgccattgccgagcgtgacacactctattcggggaatatgtcgcgtcttccgacgttagacggcgatgccgagtacttcacactctatctctcggcgcataaaattcaactgttcgagggcggcgaagcacagtccgagagtggtgaaggtgggtctgtctcatacagcactggcggcggtggcgagaaggactt |
| 18 | 55.0 | gagtgcgtccggtgtcgcggacacggagagtttgctgattgacccgcaagacgcgaagtttggcgcgatagagattcacaacatcgcccacggagggagtgttgacgtggaattgctcacgtcaagcgacgatacggaactcgtggaggatgccgctgtaacgcttgatagttttacgggcgagggaatctcacagggcaatcagattgaagcgtc |

| | | |
|---|---|---|
| | | ggataacacgaatacgtatatccgaattacgaacacgtccggcggggcgattgacattattgcgacggggcgtgaggtgtcgca |
| 19 | 56.3 | atgactgggctgaatcctgacgggttaggtcgaacggctgcatttagcaacacgtctgccgaatcggtaagtgcagtagacgcaactatcgaccgtctctacgcacaggaccgaatcgaaatccccaccgactcccgacaactgttctctactcgcgggaccgttctccgaaatttcgaggaccttagcggttggaccgccaatatcggcagtctgtcggcagagacgagcgacgtttacgtggggtcacagtcggcgcgactaaccgcctcctcgtctgctgttgacatccgttattcg |
| 20 | 53.7 | cggatacaaccactacgactatgggagctattcgattgatgagatagaggacgacgtgattcgtggcaagtacgagttgcaggatttaggcgtgagagagccgaatatcaatcactacgtctacccgagcgggaactacgctcaggagtcgattgatatgctctcgaactaccacgttatgtcgtgggggacgggagcggagtcgttcgacgccctaacgccgaatcaactgacgagtccgtggcataacctccgctgttcgttcgattcgggaaccgcagaagcggaacaggcggttaa |
| 21 | 53.3 | cgacgcggcgacgtataaccagaccgcccatatctacttccacagcgacaacgtgacgcaaagcgagatggagtcggttgcacagaccatcaactcggcggacgtgacgccgattacgctgatggacttctacaaccagcagtgacgataggatttgatgataggtttagtgagaatttcttagacgctgaccgaaaaaagacaggcgggagcaacttctttggcctgcgtgtgtccgcaaagcgagatttcattgcgggggacaaaatcgaagtgagcgcatcccaagatagcggcggc |
| 22 | 51.0 | ccagtgataccgtggtatcgtcaagcgattgctcttatcttcggcgttggagtcatgacgattgcactgtgggcaatcgaacaagggacggggccattgcttatcgggagcgttgcaggcatggcgtttctgacgattctccttatctttggcgtcgaagtcgactcgatagaagtcgatacaagcgggttcgcagtgcagttctcaaacacaacacttaccgagacaaaaaccactcgtcggagcgacaaacaccaacgggaaaatgatgatgatgattgacggggcgatttcgtggtt |
| 23 | 53.7 | gcaagcgacgggcgcaccgaactgggtggttatcgttgctattcttacccatccggcgtattgggcgaagcaactcaaaacgcgcgttagtccggttttgaatcgctatctcccaagtgagtagggcaaacgattagggttcttgtttcgttcgtgggagtatggcaacatccccagaaggcatttggtctaacagtggcgcgttgacgtttgaagacccggcagacgactcggaaatcctcttcgccggagtacgtgacgtgactatcacgcccgcgtatgaacacgccgaactctata |
| 24 | 53.7 | caacagtatcaggaaaacctcgcagagcaagaggcatttcttgagacggtggccgaggaggagggtgccgaagtcctcgaaacccagtgcaaccttatcggggagtatacggtgccactcaaagcgaaactgaatggcgaactcatggacaaactcggggagattgattcgcggattgaacacatccagagtggcgatggtcgcatgtacgagttcggcgaagtcgcagaccgggcggcacaactccttgcggacgttatcgacgacgagtcatggcataaagagaaattctatcaagcg |
| 25 | 51.0 | gcgagatgtacgactgagtgtccatacgctttaggcacgcgcgcttgaataaatgggtatgaacgccattcgtgttatcgccgggatttcagcaacgcttttcaaggtcatagccgggtttacaggcattatcgccgtctattactacgccatttgtttactcggcatgggtagccccttcgaactctatgccgcgtttctatggacaatcctgacgatgctcttcgggagtgtgtttctcgtctcggcgttccttcgtgggggtgactaatacggcttaagtgggatgccttcccattt |
| 26 | 55.0 | aaatcgacgaggacgcacgcccggcggtggggtctttcacagatgatttgtacgaggttgcagaggcaacctatgaagcagatagttcgttagaagcgtttcaaacagcgttggacggcgttaacacggcaattgacgacgtggcagtctcgacactccaaaccgaaattgaagaacttacagggattaccatcccgaagaattggcttgattttggatgggacatcatgacgcttgacgcccggcagacgatggacaatattgagacgattatcaacgagtttcccgaagacttcggga |
| 27 | 51.7 | tccgtcgtatctattcaaacgagtgagtggcggattcttttaggtttgtgggcagaatagaacagtaatggcagacacgacgatcattgacgcggttgtgttcccgcaagacgacgggacgggagtgtcgaatggagatgaagactatgatagtgccgggtatctcgcatccctcgccagatacgcgggagatggttcatatgttggtggcgattctaccggttcgccaacgcttcaattcgcaaacatcgacacggcgaatgaagaagtggacattcaaccggggcatgcgttcatcct |
| 28 | 53.3 | cgaaagtggacatattgtgcaatccgggagtcaaaaaacatacgacacgaaccttccagatagcgtcccgtatgtcgtcatcctgccgtcgagcgttacaaacgtcccgttggacacggacgttgacaacgatgtttggcttgcggttgaccccacgtctaatgactcggtttatatccggagtgggaacggcttgagtgccccaagcgacccctcggta |

| | | |
|---|---|---|
| | | aaactcggaacggttaatagttccactgggagcacaacgcgtcccaacgaccttgcagaccactcggttgatgcgctaaa |
| 29 | 50.0 | cggactcggctgacgaaatctcggtcgagaatcttagcacgacgggcagtgcggatacggtcccgataagtcaaggagatgggacattatctatgggttctattgtccccaccgacttgcaatggcgtgaggattcaaatagcccacagacagacactaacgttggcacatcaacatatacgattgcagacacgtttgacgagtacctaatccgcgtccaagtctttgaggaatcatctgctccggggacggtggagttgcgcgcggaaggcaatagtcaatcgtcatatgattatattt |
| 30 | 51.0 | ccgaagacgggacaacaacttcggcggcgacttctatcccctgcattgaattgcaagcggattcgtcaacaatatccgtgttcggcgcgtcggggagattctccgggcagtgggaatttaggtcgcaacctaacacgtcaactaatcttgcgacggcaggattcttggcagttatctcatcgccattagggagtctcgaactttttcgcgcagcgaataactttagtgtgacgtgggaagtctttggacgtgacattggcccggcaggtgttccataatgacttggacatatgaacttca |
| 31 | 53.7 | gatggcgaggagcaaaataacgtttgggatgtgcaagttgtcgacaccgcaaatccgtttggggactacgctgtgttcaaaatggacgataggggcgggcaagcatttgaagcatatccgcgtgggacgcgcgtcgaagcatatgtttcggagggaacggagccacttgacaatcggtttaccggatacgtcgtcgaacgccgagaaaacgaacaacaaggcgcggatgtacttgaggttgaagcctactcgtttgaccaattcttgcggcgcaacaccgtcacgaacgaccagacggga |
| 32 | 54.3 | ttcggcgtgggcgatgatttggagttcttcttccaaccacgcgagacagtccacattgaccggggtgtggacaacacccagtggtttagatatgacattcccgaactcgggaaagaagcaatcaacgaagtagaggtgtggtttgacgacggcgaagaaagcgtaattgtcgacgacgggacggacaaactcgacttgcaggactcactcgggttgccaagtccgggcacccaacgcaaggaattgcagcggccactcgtgactgacatttctgacgccgaagacatcgggcgcaagtat |
| 33 | 55.3 | tctcggcaaccagacggactctgtaagcgttacagagtctctgccgagtgcgaaagtcgtcgaatactcggccactctcacacagtctggcgttgaagaaatcggcttagaaacgtctacaggaacgcttctcactcgtgcgacgtttgagacgcccgttgacttgtcgagtgacacggtgacagtgacgcttaccgtctcaaatgacgatagcgtgtctcggggcgttatgaccaacgacggccaaaccgctgttcgggacgtgttggccgataactcgccgacactcccgacagacta |
| 34 | 55.3 | gctcgaactttcgttcacgcctgaacacgatattccgggagaggagtttgccctgtggtgtcggattgaaacggacttgggggggactgaccccgggccagagatcacagtcacccttgacatcgacggcgatacatactcgtgggtcccgattggcaccaataccgcgcttggcttaaactggtatgaccttgcaaacaacacgtttggagggagttcaacatacccggacaccgatattcccgaagggtccacggtcacgctcagcatagaggcaacaagttcgtccgtctcaggaca |
| 35 | 55.7 | agacggcgacgacgcggcggaacgtctcggaagcgcgattcacactcacggccaacgatacaagtgggaatttctacgtcgaacttgcgaacgacggcagcacgttcaacagagtcaacaacgccacgtccgggagtgtcacgtttgcatcgccggataccaacgtcgacaccaatatctcgctaaatcgctatgggtcgcgttcgacggcgacaccacagacggggttcaacgcacaggaaattgacaattgggaactctatgcggacattgatgctgttcttcccgacgacattggcg |
| 36 | 52.0 | cagaggcaaagcatacacattcaatcgacacgttccggagtgcgatatgccgggatgtagcaacacgacgcattggtctgaatcaatgtgcgaccaacactaaccccgttgtaagtcgggttcacaaccgaccggggttctcaggtggcgacttaccgccgtgaacacagatggacgaaagcaagaacctgaacgaatcggttgataagattcgacttcaaaccgacattagcagaggcgaaggcacccgtgaccaagaaaagcacaaactcaaggttcgtggcgagacacccgaagaag |
| 37 | 54.7 | tgactgagtgcaagatgcccggttgtaaggccgattcgcggataaaggtagacggcaagttctgttcggtcgagcaccgagtcacctacgagaaacgcgaagcagacgcccgcgaagcaatgctcgacgacaaataccccgaccgacacgaggacgaacaatgagcgaacccaaatatgccatgtacgttggcgactacgaaacgaccgtcagtgtgtggctccgaaagaacggcgaagagtatccaatctacgcgaacactcgggggcaatgtgaatggtacactcacaacgtctcgga |
| 38 | 52.7 | ccgaacatcgacatcatggaacatccagataaaattgagttgtgggtgaatctatgacggcatccgggacttatcaggagacgatggtggtctactgttgtcgaaactgtggtgccttctcgtctgtcaaaatgggcggctgcccggtgtgcgactactctgaaagcgaggagtacgaagtgaacgcattcgacggcggcacggtgacaaaaagaga |

| | | |
|---|---|---|
| | | ccgtggtacaagtctcacgaggtgctattgaagtgaccgactctaagtctgaggcacacacgtttcacgcttgc gcccggtc |
| 39 | 50.7 | tttggtcttgtcttgttgaaaggcgacccttacgagagtgaggttgttgcgcttgctggattacaccgcgtttgga cggaggaaagcaatgactgaaccaatagtcatatgcgacgattgcggacgtttcgtagaattggtgcgtatca atcgacggcttaccctgacgtgtgattgcggtgaacgacgctcggtgaaagtctcgcaagcactccctgacg ggtggcaagcatgaccgagaaattagttcggtatgaagcagtagacgacgaggcagagttagtctgttcatat gcagac |
| 40 | 54.0 | gttgacgaaggcgagaactttctcgtctgtttgaaagacgacggtcgcaagcgtcggattccaatccaccgcg ttatcatcatcgtggagaacgacccatgaccgacaaagaggacgttcggtttaacgtgattatgccgcagacg cttcgagacgacgccaagaagaacgccgaacgtggggaactctccgaagaagtccgcgacttattccggc gaaaagcctacgggatcggttcgtcagaacaaccgagtgagatagacgagacgaaagcagaactccgcga agtccgagcgcaaa |
| 41 | 52.0 | tcgacgacttacggcacaaaagaagccagatagacgcgaagatacagacgaaggaaacccgtgccgcac gacttgaagaacgcattagcgaactacaggaccaacgtgacgaagtgcaacagtcgttagacgtgttagaga atatgttacaaaacggcgaacgcatgtggccgaagcggattgaaaacgcggcagatgtcgacgaaggcac gtctcacgaactgtatcaggaactccaaacgcgaaactccgaactccctaaagcggcgtttgagcaaccaac agtcaagacaccggcgg |
| 42 | 46.0 | attggagagaagaacagagacagtagtacaatacacactacatacactatacacactacacactacacactac acactacacactacatgagcatacaacagattgaggcgtgggaagacgcatacgactacggtggcgttgttg aagcgcacgaagacgaaattcagcagttaatcaatcactacccgaaagagcagacgacactcgtgatagatt ggtccgtgatacttcgcaaggacatggacttagcagacgacgtgataacgtcgccaaacaaacacgcgaaa gcgtttgagaac |
| 43 | 54.0 | gctgttcaagacgactttggagacacacagtggacgatagaagcgggtgcgtttgtcaaggcaaacaaagg gacgtgtattatcgacgaactcgacaaggtagacggcgatgcactctcaagcctccacagtgcgcttgaaac ccaacgggtagacattgccaaagcgggaatagaaacccgactgcaatccgaaacctcactcttgggcgcgg ccaacccgacgaatgagcgatttatcggcccggagatagaatcatacgttgagcaaatcccactgggtgacg cactccgaagccgc |
| 44 | 53.7 | atggacgcggtgtttctcctcaaagacgaagttgacgaagagcgagacggcgaaatcgtcgaggctatcatc gacagtatagcgaccggggagaacgtggatacggttggcgagtgggacgattcggtacaacctgacttaga cggcgatttagtccgtgcatgggtcgcacacgaacgacaacagaaccgaatcacgatagacttagacctact taaacagcgaatcaagaagaaatacgtctcgctccggcaggcgtcaaagggaagcggagcaccaatcaac ccgcgcaaagtcccg |
| 45 | 52.7 | gcagaggacggcgacgtaatgcgtccatcgacgggcgagtatcggaggatagactaatgacgggacgca aacccaaaggcatcgtcgccttagagattcgggatgcatccgatagttacgccaaagagcgaatcaacgaga tagagcaagtctcgtatctccgggcaatcgtctcgaaagaagtcgaacgcgacaacccacggacgcaacga atcgcatggacgaaccaacgaattgcagaggtgaaagaatgagcgactacaaaccaacgataaacacaga cgaagtgccagagggaca |
| 46 | 49.0 | aaacaccattcacgttgcattagtttcgggagggatggatagcgcggtcgcagcatcggttgcatacgagac ggcaaataaaattgacttctttgcctatcttgacaccggaactggattacaagaaaacgaggattacgttaagg aactcgcacgacaccacggcgtacaaccgtggattctgggaacgcatgaagattatgaggaatgggtacaa gagcaaggatttcccggccctgcgcttcattcgacagcataccgaaaactcaaagaacggcaattggggaaa ctcgcaacgct |
| 47 | 51.3 | cgcacatccgaaagagttggttctgtggtctggcgtccgaagtgctgaatcaaagcggcgaagtctcaacgt ggaaccgatagacgacgctccacgttggaaatgggtgtcgccaatccacgattgggataaggacaaatgca aatcttaccttgatgacagggacatcccgaaaaacgaaatatgggatacactcgggcggtcgggtgattgtttc tgtggctgcttcggaaacccagaggaaaagttagacttgcgtgcgttcggagcagactaccatgccgagtgg attgaagactt |
| 48 | 52.3 | ggtgagccaaccgcgacgatagactattgcgcatatggacactacacgaagaaaccaactgacttctgggg agaacacccaccgatgacatacaaacggtgcccacatgagatacacacagacgagaatggcgtcacggac atggaacgagggccaagcgacccatccgaacgtgcaaagttaccatacggtgtttcaaataccgtattagaa |

| | | |
|---|---|---|
| | | gcgtgtgaatcggcgttagacggcaacgcgccgaaagacacgacactcacggagtggcacgaatgaccgacagcccttatgaaaaga |
| 49 | 53.3 | cgtgcccgttctgtggcgagacagtcggcagactccccgtctcacctcccgtgtgagagtgtagagggaaacgcatgagcaataacacgtgtgcacgaccgtcagacatgcacccggagtgccccgagtgtgagacggatgtgttcgtcgatgcactgtcttacgagaaagacaaataccgctgccaattctgtgaggagaactttcgggtaactcgcatctgactgttgtcccccaacacttattacaaatggggtacacagtagtagtatggaagcacacgagattgaagttggcgac |
| 50 | 54.7 | acggttcgcgtggacatcaagaacaacgctccaattggcggggcaacgcaactggcgacgttcactgtcaaagagaagtcggttaacgctatcacgggcgttgacgaatcgtggggcgatgagtgcactattagcggatggggacggattcgcttcactacaaagacggtggcaaatcgggagatattgttgaggttcgcgtgatggaaacgacagacgacggtgttccaattcacggtagcggcacggtcgttgcgtaaaccccaataggtatcgtctcggttcgagtccgagatggg |
| 51 | 53.0 | aactctgacgacgaactcacgctctcgctcggcaacaacgaaacgaaagcagtcgtctcgcgtggctacgaccaaactaatgtcgtttcgcagaacactatcaaagcaatcgacccggattcaatccgccaagagccagacattcctgaccttgagttgtcggcagcggcgacggtcgattacaagccgtttatggacgcgcttgaccacgtggttgcacacgatacaaagatgaaactcgggacggcaggcacgaatctgttgttcgggcgtgaatacacgttagggtcagcagaagta |
| 52 | 55.0 | gtgttcgacggcatcgacgtgggggttgacatcgccaagacgtggttctctggggactacgtgcaagaactccaacggatgctcaagaaagtccgaccggacacggtgacattagagtggggcgaagagatgcccctgtacgcttactttggcacagagttcgccgaatacacgttcgtcttagcaccgcgtattcagagtaactaaccctgttgtaagcgggattcacaccccgtcagggttcttatggcaaagaatggccgtctccgggtgcgaacctcggagaagaaagtccgcgaa |
| 53 | 51.0 | gtcgaaatcctgtgtgaacttctcggacaatccaagtcggagttccttcgcacggcgatagacaaccacatgcaacatcaatacctgcaactctcggaaaccgaacgccaacaggtgcaagcgaggttagacgatgccgagtgacgtagaacccgacttcagcgacgacttagaaaaaacgaacgaagcaatcaacaacgcgctaaacgaactggcgaaggtcaaagcgtacacgttgaaagtcgaagagttacgcgacttcaaagcagcacagtacgcactccgcaacatctcgccaga |
| 54 | 50.7 | aactccgagacgacgagaatcaagaggcatggatagcgtcaagcgcggggttcgtgaatctgggtgactgtgtgtgaaattcacgtatacaatggcagaggcgatgaccctcactgcaattattctcggatggatgcctacgatactggagtgggacgcgctaagcacacggtccatcgtagtttggattgtttgtgttgctttgctcgcaatcccgttcgcttatgaaaagactgtggggttgtcactatgacgtggaaagacgactatcaagacgagaaagccgagtcgggattgacg |
| 55 | 51.3 | tcgacgaactcgacgacgctacccagacgcttgcagaactaaccgagaacgtgagtgcactcatgaccgagtggaaagagacacggcgcgaactgcatgaaatcaaagtgctgctcaagagcgaggagtttgactatgagtgaattacaaacaaagcgtgagttagcgcgttctcttggactgtatcaacgacgggtcgaactacgatatgaccaattgttgcggattgaccacgagacacaatgacaccgacacagatgcacggtcgcttagacgagaaaggcctgttagacgacgcgc |
| 56 | 56.7 | cgacgacgtggagagcgtcgctcgtgcatggctccaagaagtagacgccccactttccgacaaagcgtttgaacgactctcgaatagtaaggtctgctacgtcggtgcgagcaaggatgtgtacaacaggttacaggaccatgccgaagcgagtgttcgtaaggcgttgttcttgcgcgtgttcgctcccgtggacgtagtgggtgtgtggccgtcggatagccccttccgtgatgaatactcgaaagcgttggaactttccggtgagggttgggttgcgtggtctgacggcacgttgct |
| 57 | 51.7 | ataacccgtcgacagaactaactcgacacaattcttatacaaattatggcgacttacaacggcagcacaacgtatcgtcccggatggaacgaccctcaagacgtgcaacgctggatagccgacgaactgtgtatcggaaaaacgcttgtcttcccctccgggagaacaacaattggagatgtgcgagcggacgtcgacggcgaacttgacccggacgtgatcgcagacttgcacaatcccctcgagacgtttgatgaacagtcgtttgacacggtatattgcgacccaccgtattcgttc |

**Supplemental Table S2:** Relative coverage [%] values for sense and antisense transcripts across the HFTV1 genome, based on RNA-seq data.

| **Sense transcript** | | | | | | |
|---|---|---|---|---|---|---|
| **Coverages** | **T5** | **T20** | **T60** | **T120** | **T180** | **T300** |
| TSS gp01 | 1 | 12 | 54 | 100 | 97 | 64 |
| TSS post gp02 | 0 | 3 | 11 | 100 | 92 | 58 |
| TSS gp06 | 1 | 11 | 100 | 75 | 57 | 49 |
| TSS gp06a | 9 | 67 | 100 | 40 | 22 | 17 |
| TSS gp13 | 0 | 1 | 3 | 100 | 76 | 45 |
| TSS gp14 | 0 | 1 | 7 | 100 | 92 | 98 |
| TSS non coding RNA | 0 | 4 | 21 | 59 | 100 | 54 |
| TSS gp15 | 0 | 1 | 8 | 68 | 95 | 100 |
| TSS gp18 | 0 | 0 | 2 | 82 | 100 | 57 |
| TSS gp20 | 0 | 1 | 3 | 100 | 96 | 77 |
| TSS gp21 | 1 | 12 | 33 | 100 | 61 | 26 |
| TSS gp25 | 0 | 0 | 0 | 19 | 100 | 96 |
| TSS gp26 | 1 | 2 | 15 | 96 | 93 | 100 |
| TSS gp29 | 1 | 5 | 17 | 100 | 86 | 75 |
| TSS gp30 | 0 | 1 | 7 | 100 | 100 | 62 |
| TSS gp33 | 0 | 1 | 1 | 29 | 96 | 100 |
| TSS gp35 | 0 | 1 | 8 | 100 | 65 | 41 |
| TSS gp38 | 1 | 4 | 22 | 100 | 90 | 60 |
| TSS gp39 (anti gp38) | 0 | 2 | 17 | 87 | 99 | 100 |
| TSS gp40 | 1 | 4 | 8 | 71 | 75 | 100 |
| TSS gp42 | 0 | 2 | 11 | 100 | 60 | 40 |
| TSS gp45 | 1 | 4 | 72 | 100 | 52 | 37 |
| TSS gp46a | 3 | 27 | 100 | 14 | 4 | 3 |
| TSS gp46b | 1 | 13 | 100 | 22 | 12 | 17 |
| TSS gp46 | 4 | 32 | 100 | 11 | 12 | 20 |

| | | | | | | |
|---|---|---|---|---|---|---|
| TSS gp51 | 0 | 0 | 18 | 60 | 94 | 100 |
| TSS gp52 | 4 | 60 | 100 | 24 | 21 | 19 |
| TSS post gp56 | 1 | 1 | 15 | 67 | 100 | 75 |
| TSS pre gp61 | 0 | 3 | 18 | 83 | 100 | 75 |
| TSS gp62 | 12 | 100 | 46 | 29 | 31 | 23 |
| TSS gp63 | 17 | 100 | 41 | 20 | 10 | 9 |
| TSS gp64 | 11 | 100 | 45 | 39 | 28 | 18 |
| TSS pre gp66 | 0 | 4 | 27 | 100 | 90 | 55 |
| TSS post gp66 | 0 | 0 | 11 | 100 | 93 | 74 |
| TSS gp68 | 0 | 3 | 68 | 100 | 48 | 20 |
| **Antisense transcript** | | | | | | |
| **Coverages** | **T5** | **T20** | **T60** | **T120** | **T180** | **T300** |
| TSS anti gp6a | 0 | 0 | 7 | 60 | 100 | 75 |
| TSS anti gp13 | 0 | 2 | 8 | 40 | 100 | 76 |
| TSS anti gp13 | 0 | 1 | 4 | 44 | 100 | 75 |
| TSS anti gp18 | 1 | 8 | 25 | 86 | 100 | 90 |
| TSS anti gp20 | 1 | 4 | 9 | 58 | 100 | 76 |
| TSS anti gp20 | 0 | 0 | 0 | 4 | 33 | 100 |
| TSS anti gp24 | 0 | 2 | 18 | 55 | 79 | 100 |
| TSS anti gp25 | 2 | 2 | 12 | 71 | 97 | 100 |
| TSS anti gp26 | 2 | 11 | 17 | 47 | 86 | 100 |
| TSS anti gp33 | 0 | 1 | 5 | 39 | 100 | 95 |
| TSS anti gp41 | 1 | 37 | 24 | 100 | 87 | 49 |
| TSS anti gp42 | 0 | 13 | 21 | 82 | 86 | 100 |
| TSS anti gp44 | 0 | 1 | 9 | 67 | 96 | 100 |
| TSS anti gp47 | 1 | 1 | 7 | 51 | 97 | 100 |
| TSS anti gp48 | 1 | 3 | 9 | 100 | 96 | 69 |
| TSS anti gp50 | 0 | 3 | 6 | 59 | 100 | 100 |
| TSS anti gp51 | 0 | 0 | 2 | 15 | 80 | 100 |

| | | | | | | |
|---|---|---|---|---|---|---|
| TSS anti gp56 | 0 | 0 | 3 | 59 | 100 | 56 |
| TSS anti gp 58 | 1 | 2 | 13 | 90 | 100 | 62 |
| TSS anti gp59 | 0 | 2 | 9 | 61 | 100 | 85 |
| TSS anti gp60 | 1 | 3 | 22 | 100 | 88 | 52 |
| TSS anti gp65 | 1 | 5 | 8 | 66 | 100 | 58 |

## Supplementary Figures

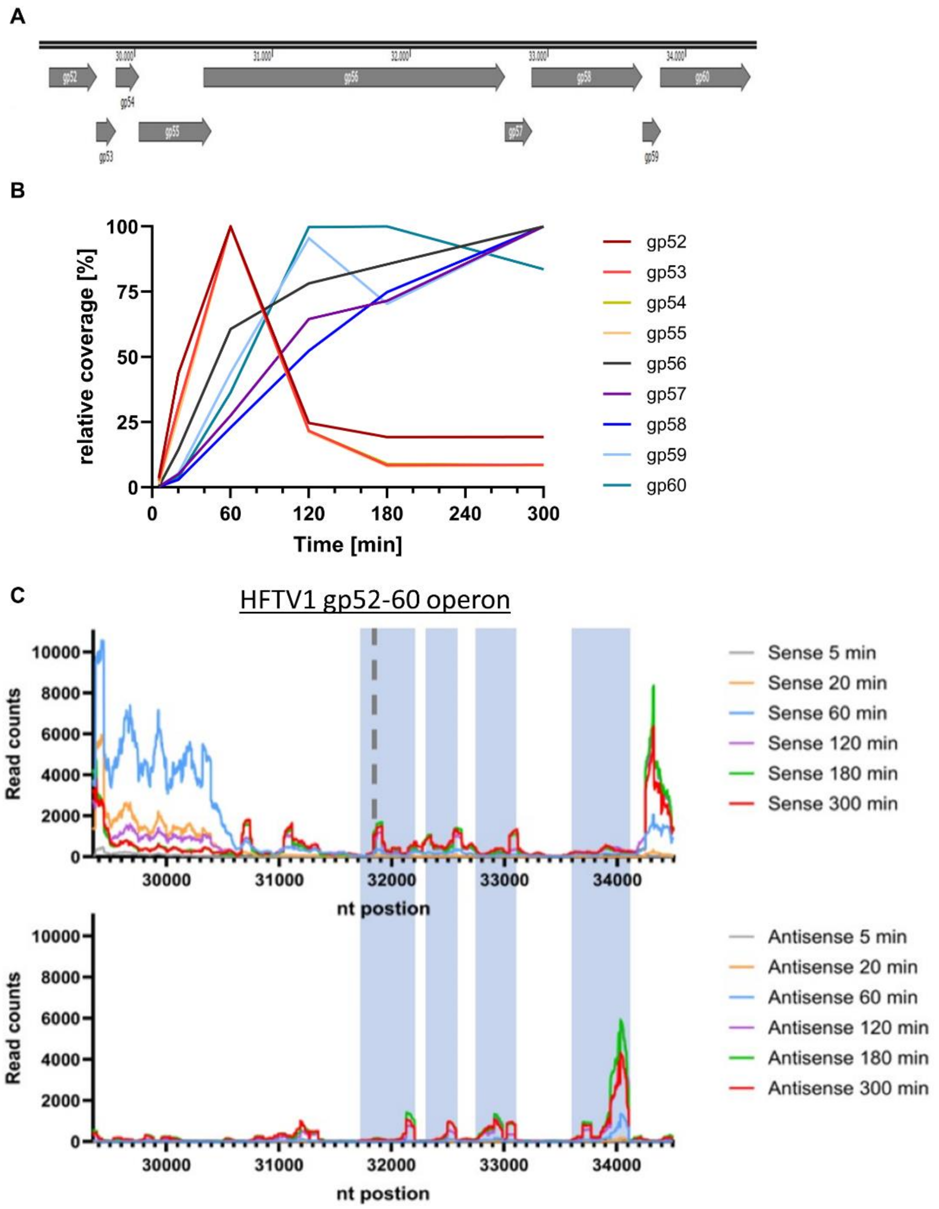


**Supplemental Figure 1**. Gene cluster structure (A) and percentage of total read counts (B), of the HFTV1 viral gene cluster and *gp52* - *gp60*. Depicted in black/grey *gp52* – *gp55* and in green *gp56* - *gp60*. (C) Read-coverage plot showing the read counts calculated as number of reads at the indicated nucleotide (nt) position in the HFTV1 genome. The vertical dashed line indicates the start of a region which has a distinctly different temporal coverage profile to the TSS at the beginning of the operon. The blue boxes indicate regions with increased antisense coverage which are preceded by a sharp increase in coverage, indicate of an antisense TSS.

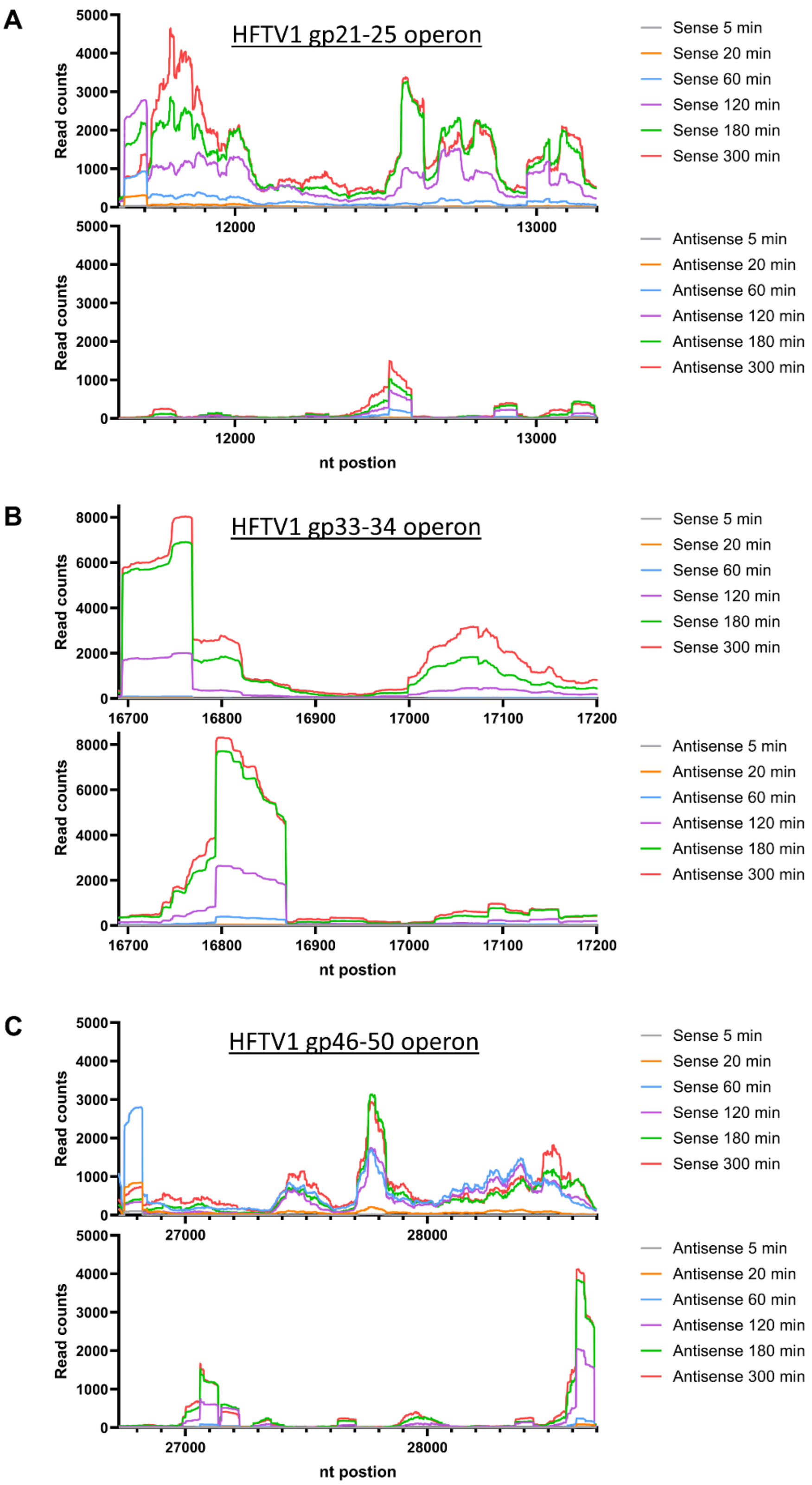


**Supplemental Figure S2:** Read -coverage plots for the operons gp21-25 (structural, A), gp33-34 (lysis, B) and gp46-50 (DNA metabolism, C) showing the read counts calculated as number of reads at the indicated nucleotide (nt) position in the HFTV1 genome.

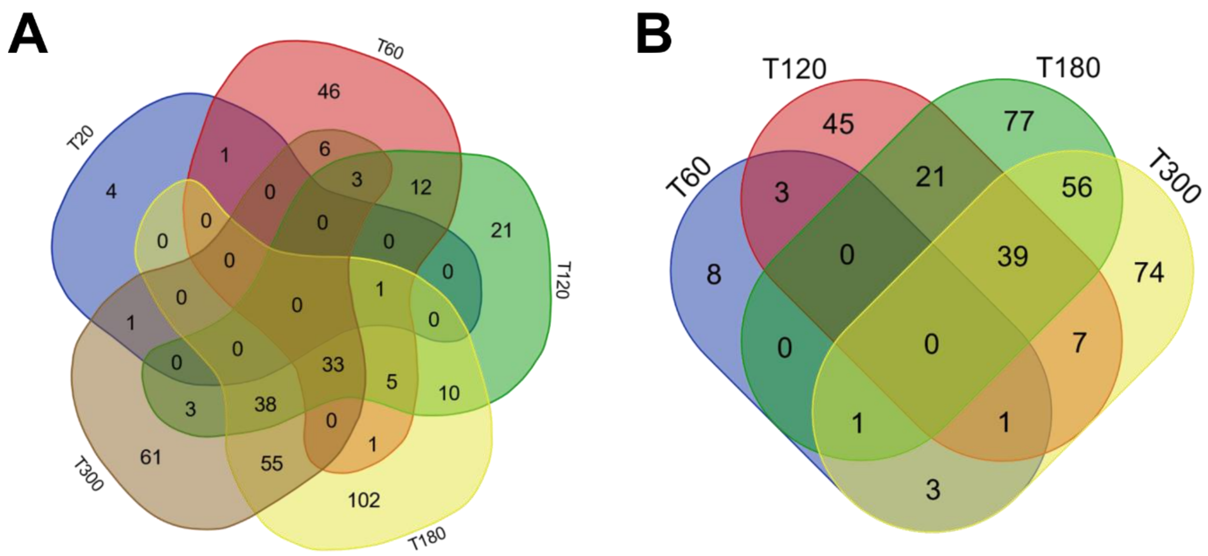


**Supplemental Figure 3:** Venn diagrams of differentially expressed genes in *H. gibbonsii* in response to HFTV1 infection. (A) Upregulated genes at 20, 60, 120, and 300 minutes post-infection and (B) Downregulated genes at 60, 120, and 300 minutes post-infection (p.i.). Each diagram represents the number of differentially expressed genes (DEGs) at each time point and their overlap, highlighting shared and unique transcriptional responses across the infection timeline.

## SUPPLEMENTARY VIDEO

**Supplemental Movie 1**. Time-lapse light microscopy of *H. gibbonsii* cells under standard conditions. Time-lapse phase-contrast microscopy of *Haloferax gibbonsii* cells under untreated conditions. Imaging was conducted at 45 °C, over a duration of 1 minute at 0.8 frames per second (fps).

**Supplemental Movie 2**. Time-lapse light microscopy of *H. gibbonsii* post HFTV1 infection. Time-lapse phase-contrast microscopy of *H. gibbonsii* 5 hours after infection with HFTV1 at a multiplicity of infection (M.O.I.) of 10. Imaging was performed at 45 °C, over a duration of 1 minute at 0.8 frames per second (fps).

# SUPPLEMENTAL REFERENCES


1. Schwarzer S, Hackl T, Oksanen HM, Quax TEF. 2023. Archaeal Host Cell Recognition and Viral Binding of HFTV1 to Its *Haloferax* Host. mBio 14:e01833-22.
2. Nuttall SD, Dyall-Smith ML. 1993. HF1 and HF2: Novel Bacteriophages of Halophilic Archaea. Virology 197:678-684.
3. Moraru C. 2021. Gene-PROBER - a tool to design polynucleotide probes for targeting microbial genes. Syst Appl Microbiol 44:126173.
4. Barrero-Canosa J, Moraru C. 2021. Linking Microbes to Their Genes at Single Cell Level with Direct-geneFISH, p 169-205. *In* Azevedo NF, Almeida C (ed), Fluorescence In-Situ Hybridization (FISH) for Microbial Cells: Methods and Concepts doi:10.1007/978-1-0716-1115-9_12. Springer US, New York, NY.
5. Bouras G, Nepal R, Houtak G, Psaltis AJ, Wormald P-J, Vreugde S. 2022. Pharokka: a fast scalable bacteriophage annotation tool. Bioinformatics 39.
6. McNair K, Zhou C, Dinsdale EA, Souza B, Edwards RA. 2019. PHANOTATE: a novel approach to gene identification in phage genomes. Bioinformatics 35:4537-4542.
7. Chan Patricia P, Lin Brian Y, Mak Allysia J, Lowe Todd M. 2021. tRNAscan-SE 2.0: improved detection and functional classification of transfer RNA genes. Nucleic Acids Research 49:9077-9096.
8. Terzian P, Olo Ndela E, Galiez C, Lossouarn J, Pérez Bucio Rubén E, Mom R, Toussaint A, Petit M-A, Enault F. 2021. PHROG: families of prokaryotic virus proteins clustered using remote homology. NAR Genomics and Bioinformatics 3.
9. Chen L, Yang J, Yu J, Yao Z, Sun L, Shen Y, Jin Q. 2005. VFDB: a reference database for bacterial virulence factors. Nucleic Acids Research 33:D325-D328.
10. Alcock BP, Raphenya AR, Lau TTY, Tsang KK, Bouchard M, Edalatmand A, Huynh W, Nguyen A-LV, Cheng AA, Liu S, Min SY, Miroshnichenko A, Tran H-K, Werfalli RE, Nasir JA, Oloni M, Speicher DJ, Florescu A, Singh B, Faltyn M, Hernandez-Koutoucheva A, Sharma AN, Bordeleau E, Pawlowski AC, Zubyk HL, Dooley D, Griffiths E, Maguire F, Winsor GL, Beiko RG, Brinkman FSL, Hsiao WWL, Domselaar GV, McArthur AG. 2019. CARD 2020: antibiotic resistome surveillance with the comprehensive antibiotic resistance database. Nucleic Acids Research 48:D517-D525.
11. Steinegger M, Söding J. 2017. MMseqs2 enables sensitive protein sequence searching for the analysis of massive data sets. Nature Biotechnology 35:1026-1028.
12. Ondov BD, Treangen TJ, Melsted P, Mallonee AB, Bergman NH, Koren S, Phillippy AM. 2016. Mash: fast genome and metagenome distance estimation using MinHash. Genome Biology 17:132.
13. Cook R, Brown N, Redgwell T, Rihtman B, Barnes M, Clokie M, Stekel DJ, Hobman J, Jones MA, Millard A. 2021. INfrastructure for a PHAge REference Database: Identification of Large-Scale Biases in the Current Collection of Cultured Phage Genomes. PHAGE 2:214-223.
14. Heinzinger M, Weissenow K, Sanchez Joaquin G, Henkel A, Mirdita M, Steinegger M, Rost B. 2024. Bilingual language model for protein sequence and structure. NAR Genomics and Bioinformatics 6.
15. van Kempen M, Kim SS, Tumescheit C, Mirdita M, Lee J, Gilchrist CLM, Söding J, Steinegger M. 2024. Fast and accurate protein structure search with Foldseek. Nature Biotechnology 42:243-246.
16. Ferla MP, Pagnamenta AT, Damerell D, Taylor JC, Marsden BD. 2020. MichelaNglo: sculpting protein views on web pages without coding. Bioinformatics 36:3268-3270.
